# A General Framework of Persistence Strategies for Biological Systems Helps Explain Domains of Life

**DOI:** 10.3389/fgene.2013.00016

**Published:** 2013-02-25

**Authors:** Liudmila S. Yafremava, Monica Wielgos, Suravi Thomas, Arshan Nasir, Minglei Wang, Jay E. Mittenthal, Gustavo Caetano-Anollés

**Affiliations:** ^1^Evolutionary Bioinformatics Laboratory, Department of Crop Sciences, University of IllinoisUrbana, IL, USA; ^2^School of Molecular and Cell Biology, University of IllinoisUrbana, IL, USA; ^3^Department of Cell and Developmental Biology, University of IllinoisUrbana, IL, USA

**Keywords:** economy, flexibility, gap, proteome evolution, redundancy, robustness, scope, umwelt

## Abstract

The nature and cause of the division of organisms in superkingdoms is not fully understood. Assuming that environment shapes physiology, here we construct a novel theoretical framework that helps identify general patterns of organism persistence. This framework is based on Jacob von Uexküll’s organism-centric view of the environment and James G. Miller’s view of organisms as matter-energy-information processing molecular machines. Three concepts describe an organism’s environmental niche: scope, umwelt, and gap. Scope denotes the entirety of environmental events and conditions to which the organism is exposed during its lifetime. Umwelt encompasses an organism’s perception of these events. The gap is the organism’s blind spot, the scope that is not covered by umwelt. These concepts bring organisms of different complexity to a common ecological denominator. Ecological and physiological data suggest organisms persist using three strategies: flexibility, robustness, and economy. All organisms use umwelt information to flexibly adapt to environmental change. They implement robustness against environmental perturbations within the gap generally through redundancy and reliability of internal constituents. Both flexibility and robustness improve survival. However, they also incur metabolic matter-energy processing costs, which otherwise could have been used for growth and reproduction. Lineages evolve unique tradeoff solutions among strategies in the space of what we call “a persistence triangle.” Protein domain architecture and other evidence support the preferential use of flexibility and robustness properties. Archaea and Bacteria gravitate toward the triangle’s economy vertex, with Archaea biased toward robustness. Eukarya trade economy for survivability. Protista occupy a saddle manifold separating akaryotes from multicellular organisms. Plants and the more flexible Fungi share an economic stratum, and Metazoa are locked in a positive feedback loop toward flexibility.

## Introduction

The division of cellular organisms into six kingdoms (Whittaker, 1969) and three superkingdoms has been confirmed by a wide variety of means (e.g., Gogarten and Taiz, 1992; Pace, 1997; Ciccarelli et al., 2006; Wang and Caetano-Anollés, 2006; Ding et al., 2008; Kim and Caetano-Anollés, 2012). Still, we do not completely understand the nature of the fundamental differences between them (e.g., Woese, 1987, 2002; Koch, 1998; Cavalier-Smith, 2002a; Horiike et al., 2002; Kurland et al., 2006; Valentine, 2007; among many others). Genetic sequences, molecular organization, morphology, nutrition, ecological preferences, and adaptations have been used either separately or in combinations to resolve groups of organisms and explain their differences. While our comparative knowledge becomes more detailed over time, it would be useful to synthesize the essence of what makes an organism or lineage unique. The theory of evolution by natural selection has provided one framework for such synthesis: organisms persist in an environment by evolving internal organization that works in that environment. By definition, lineages of the six kingdoms have demonstrated persistence, and a large catalog of their idiosyncratic environmental adaptations exists in the literature. Can we now design a framework that will help us describe their *method* of persistence, and express what makes one organism different from another, in those terms?

To accomplish this goal, we need a language for comparative inquiry. Miller’s theory of living systems (Miller, 1995) is a well-established theoretical framework that provides such language. It identifies the entire set of 20 subsystems required for operation of any living system, and all the relationships between those subsystems. It is therefore complete, as the 20 subsystems are necessary and sufficient to describe any living system at any level of complexity. It scales perfectly across the levels of complexity because it describes all life, from cells to societies, in exactly the same way. It works well, as the internal subsystems and their relationships have been substantiated in great detail. Thus, Miller’s framework is a good choice for our problem. The framework describes the interaction of organisms with their environment as ingestion, processing, and extrusion of matter, energy, and information. We could start by comparing organisms in terms of the magnitude and strategies of that processing.

Processing of matter and energy has been considered in a number of life history theories (e.g., Pianka, 1972; Taylor et al., 1990; Mueller and Diamond, 2001; Brown et al., 2004; Seibel and Drazen, 2007). To compare information processing between organisms, environments must be described in a comparable way. We find that the organism-centric model of the environment, pioneered by von Uexküll (1909) for Metazoa, is suitable. The model consists of describing an organism’s environment via the so-called “umwelt,” literally “the world around us.” Umwelt refers to the entirety of an organism’s perception, and therefore accounts for all environmental signals (information) that are processed by the organism. By representing the environment as the organism perceives it, at its own spatio-temporal scale, umwelt makes it possible to compare environments for very different organisms. However, organisms also evolve methods to withstand signals they do not process. For example, the outer layer of tree bark is essentially dead tissue, which serves to protect it against some external influences without processing and responding to them. Thus, the concept of umwelt is not sufficient to describe an organism’s environment, which leads us to introduce two new concepts – scope and gap.

**Figure 1:**
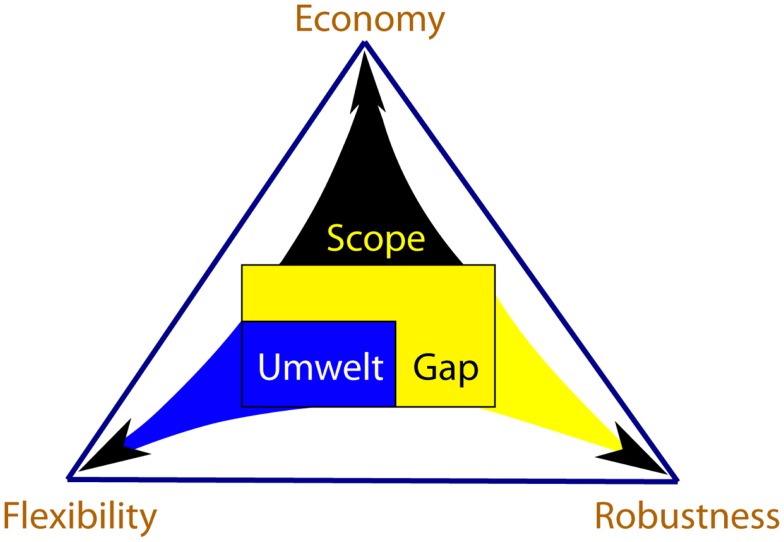
**The persistence triangle depicts the set of solutions to the tradeoff problem among economy, flexibility and robustness**. The elements of Scope/Umwelt/Gap trio and Economy/Flexibility/Robustness trio are intimately related, reflecting the effect environment has on evolution of an organism’s persistence strategies. Scope size is correlated with the organism’s budget, giving rise to the strategy of economy. Umwelt is composed of scope signals, which are processed by the organism, making it more flexible in response to the environment. Organisms evolve properties of robustness against the gap signals, which are not processed. Mechanisms of flexibility and robustness both cost matter-energy and compete with each other for the budget quota. Thus, the three persistence strategies are in a tradeoff relationship. Each location within the persistence triangle denotes a solution to this tradeoff problem.

We use the term “scope” to denote the entirety of signals to which the organism is exposed during its lifetime. Because processing signals is costly, organisms perceive and respond only to a fraction of their scope, called umwelt. Responses of an organism to the signals of the umwelt help modulate the environmental effects on the organism’s function, making the organism more flexible. The signals that are not perceived and processed by the organism fall into the “gap” between scope and umwelt. Organisms evolve properties of robustness, which allow them to continue functioning despite possible effects of the gap signals. The costs associated with flexibility and robustness are offset by the organism’s matter-energy budget. Flexible responses and robustness properties compete for this budget and are thus in a tradeoff relationship, resulting in evolution of a particular economy – a method of meeting the organism’s budget. Lineages evolve unique tradeoff solutions among economy, flexibility and robustness, and the space of all those solutions forms what we call the persistence triangle (Figure 1). The coevolving economy/flexibility/robustness trio is thus a dynamic attribute of every lineage, describing its particular strategy of persistence.

We studied the patterns of division of scope into umwelt and gap for lineages that are extant, attempting to understand what flexibility/robustness tradeoffs they adopt. We find that the six kingdoms break up into four economic strata, in order of increasing budget: Archaea-Bacteria, Protista, Fungi-Plants, and Metazoa. Kingdoms that share a stratum are resolved based on their persistence tradeoff: Bacteria are more flexible and less robust than Archaea. Fungi are more flexible and less robust than Plants. Metazoa invest primarily into flexibility. We demonstrate that the trio of economy/flexibility/robustness is a complete set of persistence strategies. It is sufficient to adequately describe an organism’s method of persistence, and compare it to that of another organism, regardless of the level of complexity. We also find that some features of the organism’s internal organization, such as diversity, redundancy, modularity, combinatorial use, and reuse of internal parts are correlated with the tradeoff biases. We hypothesize that these features constrain the evolution of lineages toward particular persistence strategies.

In the following sections, we first define key terminology of our framework. We then use ecological and physiological data to identify tendencies of organisms in scope/umwelt/gap and economy/flexibility/robustness. These data permit us to identify economy, flexibility, and robustness as the strategies of persistence and formulate hypotheses about the tradeoffs between them. Lastly, we describe coevolution of persistence strategies and coevolutionary constraints.

## Description of the Framework

Life is manifested as persistent lineages of mortal self-replicating individuals that compete with each other for resources, striving to survive, grow, and reproduce. The distinctive features of each lineage evolved through the experience of all generations to-date. To avoid confusion, we use the terms “organism” or “organism kind” for sets of lineages with similar characteristics, but also discuss individuals, where appropriate.

We adopt Miller’s theory of living systems, which models organisms as a class of emergent dissipative systems, i.e., as engines that organize their own structure by using energy from gradients of electro-chemical potential and of radiant energy (Morowitz and Smith, 2007). The theory captures this by representing an individual organism as a network of molecular machines, which take in, process and extrude matter, energy, and information. Because matter and energy are inter-convertible, we follow Miller in referring to them both jointly as matter-energy (Table 1). Information arises from spatio-temporal inhomogeneity (Umpleby, 2007) in an individual’s external and internal environment. Information is used by individual organisms for homeostasis, to function and replicate, to avoid the risk of adverse events, to locate resources, and to communicate. Matter-energy is used to manufacture the required components and to process information. We conjecture that the fundamental differences between organisms could be captured in the ways they process matter-energy and information. This may exhibit itself as processing different amounts of matter-energy and/or information, using different mechanisms for processing, or performing processing at different rates. Our objective is to find an adequate abstract representation of those differences through synthesis of ecological and physiological knowledge at all levels of complexity.

**Table 1:** **Elaboration on the fundamental concepts**.

Concept	Definition
Matter-energy	In his theory of living systems, Miller (1995) refers to matter and energy jointly in order to follow the principle of mass-energy equivalence established in physics. This principle may seem remote from the problems of biology. Nonetheless, the joint term of matter-energy is valid for living systems, because for biological organisms matter and energy are biochemically inseparable. Interconversions of energy during chemical reactions alter the underlying chemicals (particles of matter), and are fundamental to the process of life. The term is therefore useful for capturing the total flux of enthalpy through an organism
Information	Matter-energy and information are related. Information is always borne on a material marker. Distribution of matter-energy in the environment is inhomogeneous, and thus embeds information. An organism’s material components interact with each other, communicating and changing their states, which is a form of information flow. Thus, in organisms the fluxes of matter-energy and information are like two sides of a coin: somewhat different, but not entirely separable.
	Information arises from the environmental inhomogeneity. Thus, the spatial and temporal variability in ecological niches are literally measured by the amount on information to which the organism is exposed. Because these concepts are very abstract, we choose to express the organism’s environment in terms of signals
Signal	This term is used to describe what is happening in the external environment as well as within an individual organism. Depending on the context, signals may describe physical events and quantities, values of physical quantities, as well as patterns formed by values of physical quantities. Examples of signals include chemical: nutrients, pH, salinity, moisture, etc.; physical: temperature, pressure, illumination, etc.; social: proximity to and signals issued by other organisms, etc.
Flexibility mechanisms	These mechanisms are expressed either through inner changes or through outward behaviors. Examples include: inner changes: gene expression patterns, intracellular signaling cascades, heart rate modulation, stomach juice secretion, melatonin production, subcutaneous fat accumulation and loss, learning, etc.; outward behaviors: movement of any kind, taxis, ingestion, egestion, pheromone secretion, leaf/tail shedding, etc. An organism is considered more flexible if it is able to respond with a greater number of inner changes or outward behaviors to a greater number of informative patterns in its environment. The latter can be represented by a greater number of different physical quantities, or by a broader range of values of the same physical quantity, or by greater complexity of spatio-temporal patterns formed by the values of a physical quantity. In the text, all of these representations are referred to as “signals”
Robustness properties	Features and properties that make an organism less vulnerable with respect to signals it cannot process. Examples include: cell wall, bark and thorns, skin, fur, horns, teeth, shells, claws and skeleton, constitutively produced poison or other chemical repellant, thermophilic proteins, etc. An organism is considered more robust if it is able to withstand without change a greater number of informative patterns (signals) in its environment

Scope defines the spatio-temporal parcel of the environment that the individual is exposed to, and therefore the amount of information and matter-energy available to it. The term “scope” literally means “outlook,” or “extent of view.” Every individual lives within its own spatio-temporal scale, from which it scans, “scopes out,” the environment, as if through a window. The temporal dimension of scope is circumscribed by the individual’s life span. For example, in a lichen colony covering a rock, a single cyanobacterium that comes into being on a summer day may never experience the cold of winter, because its lifetime is much shorter than the time-scale of temporal variation of the seasons. Within its narrow temporal scope it experiences environmental temperature as being fairly constant. In contrast, the snake living under the same rock is exposed to a more variable environment, as its temporal scale encompasses that of the seasons. The spatial dimension of scope is circumscribed by the individual’s body size, surface area, and motility range, as these parameters determine the amount of the exposure of the individual to the environment. The amount of matter-energy that is processed increases with body size and motility. Thus, scope size is positively related to the amount of matter-energy that is processed by the individual (Figure 1), while the signal content of the scope (Table 1) constrains its information flux. Two organisms with the same scope size may be exposed to different amount of information depending on how quickly their environmental signals change. For example, plant individuals may be exposed to a similar range of temperatures in a dry desert and in a temperate climate, with the total amplitude of variation being tens of degrees centigrade. However, the former experience this variation diurnally, whereas the latter experience it seasonally. We call an organism’s scope “dense” when individuals are exposed to external environmental signals that change rapidly at the individual’s temporal scale, with amplitude that encompasses signal values that have different effects on the organism. This may be due to the environment being highly in homogeneous in space-time, or due to the individuals exploring the environment at high speed.

The scope changes as the individual goes through different life stages, such as egg, larval instar, pupa, and adult stages of a butterfly development. The scope of an individual does not have to include all environmental signals to which its lineage may be exposed. In our example above, a single cyanobacterium is exposed to different temperatures during the day or at night, but the lineage at large is exposed to the entire diurnal temperature range.

Individuals process some of the information to which they are exposed, by perceiving and responding to signals with inner changes or outward behaviors. Those signals comprise a part of the scope, traditionally termed umwelt, which describes the world of an organism’s perception (von Uexküll, 1909). An individual’s responses to umwelt signals enable it to flexibly adapt to its environment, maintaining the physical, developmental, physiological, and social parameters within ranges conducive to persistence of its lineage. For example, cyanobacteria in the ocean surface switch from phototrophy to chemorganotrophy as mixing moves them into darker waters (external signal causing inner changes), and the pit viper attacks a prey in response to the infrared radiation emitted by it (external signal causing outward behaviors). Umwelt does not describe the meaning of information carried by the signals – that is the subject of semiotics (Barbieri, 2008), and we leave it out of this paper.

Processing signals is costly: the respective molecular machinery is made of matter and requires energy to function. The signals that the organism fails to process fall into “the gap” between the experienced world and the perceived world: between scope and umwelt. By definition, the signals and events that happen in the gap do not elicit any responses from the individual exposed to them, regardless of the effect they may have on its fitness, including its survival and reproduction. Organisms evolve properties that allow individuals to continue functioning despite the effects of gap signals, and therefore make individuals more robust to those signals, at the cost of missed opportunities when information contained in the signal is ignored. These robustness properties include reliability and redundancy of an individual’s internal machinery, or protective shielding. For example, an enzyme that maintains its catalytic activity (is reliable) over a broad range of temperatures is robust against deviations from its optimum temperature of operation. An organism that ventures into a variety of thermal environments can keep using this enzyme (robustness) and continue functioning without switching to a different one (flexibility).

The distinction between flexibility and robustness is tricky. Robustness is frequently used in the literature to refer to any mechanism that allows maintenance of function despite external influence. For example, robust operation of an airport involves maintaining a schedule of flights despite malfunctions of equipment. Detecting the malfunctions and effecting timely repairs, or replacing the equipment with functional copies may accomplish this. Detection, repair, and replacement are actions that reflect changes within the system and require processing of information. We call them mechanisms of flexibility. On the other hand, robustness of a bridge against physical strain imposed by the load is a passive property, which allows the bridge to function without processing information about that load. We use the term robustness in this passive sense. More examples of flexibility mechanisms and robustness properties are given in Table 1.

In the next section, we attempt to compare organisms in different kingdoms and superkingdoms in terms of their flexibility and robustness. What does it mean when an organism is more flexible, or more robust? Flexibility and robustness are defined in terms of the division of scope signals into umwelt and gap. Thus, the greater is the range of signal values that the organism can withstand without having to change itself, the more robust the organism is against that signal. For example, all else being equal, an animal with thicker bones is more robust against forces of physical stress and deformation than an animal with thinner bones. Similarly, the greater is the number of responses generated by an organism to different values of the same signal, the more flexible is the organism with respect to that signal. For example, mammals have a wide array of behaviors in response to the different patterns of intensity and color of the experienced illumination, whereas bacteria at best can tell light from dark. At this point, it is obvious that the division of scope into umwelt and gap is exclusive: either a signal is processed by the organism, or it is not. However, it is not clear whether flexibility and robustness are exclusive. If one organism is more flexible than another, is it also less robust? We will return to this point later in the paper.

## Evidential Support of the Framework

Organisms in the six kingdoms exhibit easily recognizable patterns of scope, budget, flexibility, and robustness. We demonstrate these patterns based on the data collected from the literature.

### Scope sizes and matter-energy budgets of organisms

**Figure 2:**
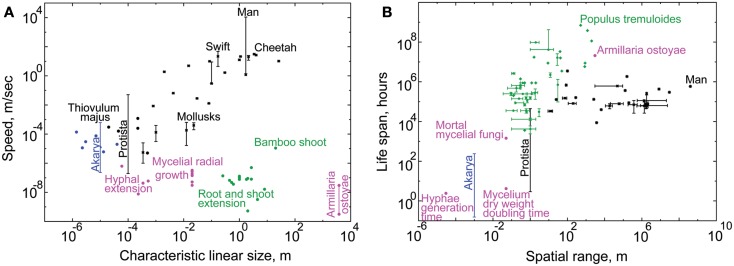
**The tendencies for scope size evolution in the six kingdoms**. In our theory, linear size and spatial range of individuals model spatial scope size. Life span models temporal scope size. By plotting these variables one against another, we demonstrate the differences in scope sizes among organismal kingdoms. Blue dots mark akarya, black dots – Protista, magenta dots – Fungi, green dots – Plants, and black stars – Metazoa. Vertical and horizontal lines indicate known ranges, not standard errors (see Datasheet S1 in Supplementary Material). Each datapoint displays measurements characteristic to individuals of a species, except for fungal data, which emphasize the spatio-temporal ranges of hyphae and mycelia (see text for explanation). It is notoriously difficult to measure the linear size and spatial range of individuals for some kingdoms, and their horizontal location on the graph should be taken with a grain of salt (see Text and Methods in Datasheet S4 in Supplementary Material). **(A)** This graph emphasizes the different strategies of spatial scope size evolution between Metazoa and Fungi/Plants. Sessile Plants and Fungi can grow to very large sizes (up to the largest known fungal genet of *Armillaria* *ostoyae*); while Metazoa achieve great motility speeds (man is the fastest). *Thiovulum majus* on top of the akaryal bar marks the fastest known bacterial species. **(B)** This graph demonstrates the different emphases in evolution of temporal and spatial scope size between Metazoa and Fungi/Plants. The kingdoms of Plants and Fungi evolve individuals with very long life spans (up to the longest-living known plant genet of *Populus tremuloides*), while Metazoa evolve individuals with very broad spatial ranges (man travels the farthest). Together parts **(A,B)** of this figure show the differences in tendencies for scope size evolution between Metazoa and Plants/Fungi: the former evolve wider spatial scope, while the latter evolve wider temporal scope, relative to one another. See Datasheet S4 in Supplementary Material for Methods.

**Figure 3:**
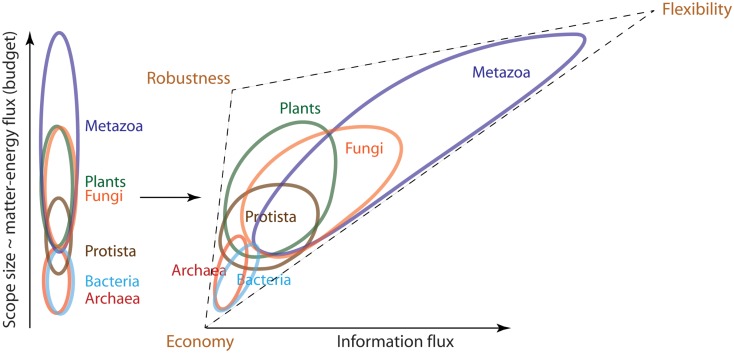
**Organisms with a particular propensity toward greater economy, flexibility or robustness tend to segregate based on the sizes of matter-energy and information fluxes they process**. The left panel of this cartoon shows that organisms segregate along the budgetary axis in the order of Archaea-Bacteria < Protista < Plants-Fungi < Metazoa. Once we add the information flux axis on the right panel, this segregation transforms into a triangle that resolves Archaea from Bacteria and Plants from Fungi based on their propensity toward robustness and flexibility. The very elongated Metazoan cloud arises from their trademark tendency to evolve lifestyles of traversing environmental gradients at high speeds. The resultant large and dense scope, coupled with principally predatory nutrition, requires that Metazoa process large information fluxes (have large umwelt). The size of umwelt progressively increases for the more evolved Metazoa, culminating in Man, who expands his own sensory and processing capabilities through manufacture, including production of sensors and computers.

Scope size measures how much of the external environment the individual can scan, or how many external signals it can potentially be exposed to. This exposure grows with the individual’s size, spatial range due to motility, and life span. The plots in Figure 2 recapitulate the known positive relationship between these variables (Harestad and Bunnell, 1979; Jenkins, 1981; Garland, 1983; McMahon and Bonner, 1983; Reich, 2001; Hedenstrom, 2003; Speakman, 2005). Based on these data and the factors contributing to budget, we arranged the six kingdoms in order of increasing scope size and budget (Figure 3, left panel). Motility and nutrition appear to be the defining factors in this distribution.

The microbes of kingdoms Bacteria and Archaea, which we here denote collectively as “Akarya,” are the smallest and slowest organisms among the six kingdoms. Motility is costly, inefficient, and simply not very useful at their spatial scale (Berg and Purcell, 1977; Purcell, 1977), due to the substantial viscous drag (Berg, 1993) and susceptibility to the disorienting rotational diffusion caused by the Brownian motion (Berg, 1993). The minuscule cell sizes and speeds (Figure 2A) result in equally minuscule characteristic spatial scope and lifespan (Figure 3, left, bottom of the axis). Protista include a wide range of organisms, some as small as akaryal microbes (e.g., *Ostreococcus*) and sessile, others orders of magnitude bigger and farther ranging than the biggest and fastest akarya (Datasheet S1 in Supplementary Material). Protista are better swimmers than Akarya, because their size makes them more resistant to Brownian disorientation. Their speeds (Datasheet S1 in Supplementary Material) are sufficiently high to attain Reynold’s numbers of ∼1, balancing the contributions of viscosity and inertia (Purcell, 1977). However, viscous drag also increases with cell size, making motility more expensive for Protista than for Akarya (Crawford, 1992). Consequently, protists have higher cell-specific (Glazier, 2009; Johnson et al., 2009), and mass-specific (Makarieva et al., 2008) metabolic rate compared to akaryotic microbes, encouraging bigger budget. Thus, Protista overlap with Akarya in terms of scope size, but extend far beyond them into the areas of bigger budget (Figure 3, left).

A multicellular organism’s budget by definition includes, and is greater than, the individual budget of each unicell comprising it. Multicellular kingdoms are very diverse, and overlap with unicells in terms of scope size. For example, the multicellular *Volvox* colonies are ∼500 μm in diameter (Sharma, 2002), whereas the bacterium *Thiomargarita* *namibiensis* is ∼100–750 μm (Schulz and Jorgensen, 2001). Yet the extent of this overlap is negligible compared to the maximum scope size achievable by fungi, plants, and animals. The largest and longest-living organisms have evolved within Plants and Fungi (Figure 2). However, overall they tend to have smaller budgets than Metazoa. First, they are sessile, which means they do not incur the high metabolic costs of motility characteristic of the highly mobile and far-ranging Metazoa (Datasheet S1 in Supplementary Material). Second, vacuoles can occupy up to 80% of cell volume in Plants and Fungi, yet they comprise a very small fraction of a cell’s metabolic budget (Miller, 1938; Klionsky et al., 1990; Veses et al., 2008), further reducing the metabolic costs. Third, Plants and Fungi adopt extended shapes of thin flat sheets and long filaments, which accommodate their absorptive mode of nutrient consumption (as opposed to the metazoan engulfment). This means they can span the same space and have the same surface area but less mass, compared to animals. Smaller mass concurrently results in smaller metabolic cost for the same body exposure to the elements, and therefore smaller budget. Additionally, the absorptive consumption creates the problem of local nutrient depletion in the environment immediately adjacent to a hypha or a root, which can limit the rate of matter-energy influx down to the rate of diffusion. Finally, the nutrients consumed by Plants and Fungi do not tend to yield as much matter-energy as those consumed by Metazoa. Fungi lead saprotrophic lifestyles, spending significant time within oxygen-poor substrates. These organisms make extensive use of fermentation, which is considerably less efficient than aerobic respiration characteristic of Metazoa (White, 2000). Plant photosynthesis is efficient compared to fermentation and aerobic respiration (Allen et al., 2011), but has a low yield per nutrient particle. Productivity is higher, the greater the influx of photons. Indeed, the incident number of light particles per unit area of an individual can be much higher compared to glucose, due to its molecular size and environmental distribution. However, this does not significantly improve nutrient availability for plants. First, only a fraction of total spectrum of solar radiation is photosynthetically active. Second, illuminance is subject to considerable variation annually (Papaioannou et al., 1993; Byun and Cho, 2006), diurnally (Wang et al., 2005), and due to cloudiness and overshadowing by objects (Young and Smith, 1983). Thus, while plants regularly receive some light every day, the exact amount can be highly variable, from fairly little (e.g., in forest understory) to saturation. The matter-energy flux achievable by plants is also constrained by the fact that their various nutrients are not co-localized: light and CO_2_ must be obtained from above ground, but water and minerals from below ground. Combining all these factors together, we placed the kingdoms of Plants and Fungi in the budgetary stratum between Protista and Metazoa (Figure 3, left).

Metazoan nutrition consists, with very rare exceptions (Pierce et al., 1996; Danovaro et al., 2010), exclusively of other organisms, be they live (predation, grazing), or dead (saprotrophy). Predation provides the greatest matter-energy yield using the known biochemical pathways. First, large amounts of energy can be extracted efficiently using aerobic respiration on organic material (White, 2000). Second, organic substances can simultaneously serve as sources of carbon and energy. Finally, Metazoa engulf food in large quantities, sometimes much bigger than their own body (e.g., boa constrictors, Owen, 1866). Consequently, Metazoa tend to process large fluxes of matter-energy, as evidenced by their nutrition alone. Many other factors associated with predatory lifestyle increase budgets of Metazoa far beyond those of other kingdoms. Speed and endurance (useful for both predators and prey) are determined by muscle performance, which is temperature-dependent. Homeothermy quite possibly evolved to provide the thermal conditions necessary for better muscle performance (Bennett, 1985; Carrier, 1987), but is maintained at high metabolic cost (Bennett and Ruben, 1979; Robinson et al., 1983). In addition to elevated temperature, increased running speed requires elevated oxygen consumption (McMahon and Bonner, 1983). Greater mass in mammals permits larger mitochondrial and capillary volumes, which improves oxygen supply to locomotory musculature (McMahon and Bonner, 1983; Weibel et al., 2004), at the cost of higher metabolic rate. Finally, the skeleton is essential for maintenance of high motility speeds, as it provides attachment points for muscles, stores elastic energy, transmits forces from the limbs, and helps ventilate lungs (Koob and Long, 2000). The stress imposed by motile activities upon the skeleton results in structural costs. This stress is reflected even in the chemical composition of the skeleton, which in vertebrates evolved to be dominated by calcium phosphate (instead of calcium carbonate used by the rest of Metazoa) to withstand dissolution due to systemic acidosis caused by intense muscular activity (Ruben and Bennet, 1987).

We can represent kingdoms in a cartoon form as clouds of different lineages that comprise them, aligned in order of increasing budget (Figure 3, left). In this representation, the kingdoms form overlapping strata along the budgetary axis, supported by the data on metabolic rate, structural costs, spatial scope size, and lifespan. Some kingdoms remain unresolved within their strata, such that they appear in the following order: Archaea-Bacteria < Protista < Fungi-Plants < Metazoa.

### Patterns of flexibility

Short of enumerating all the flexibility mechanisms, it would be difficult to prove that one organism is more flexible than another. However, we defined relative flexibility by the number of signals an organism can use to exploit, avoid or adapt to an environmental change. Thus, we can make a reasonable case by integrating the comparisons of scope content characteristic of organisms in each kingdom, with the most obvious features of flexibility. We illustrate this in Figure 3 (right panel) by adding the information processing dimension to the budgetary axis.

Akarya clearly have the smallest scope size. Most environmental signals vary at spatio-temporal scales significantly greater than that of the Akarya and thus appear constant through their scope window. Social and nutritive chemical signals are an important exception: akarya can detect binding of a single molecule, and alter their behavior numerous times throughout the life depending on the chemical signals they receive. The most obvious difference between bacterial and archaeal scope content seems to lie in their nutrition. Archaea prefer energy-stressed environments (Valentine, 2007), where matter-energy yield of nutrients is low and growth is slow. Bacteria are present in almost all niches where Archaea are present, but not the other way around. Many bacterial species are exposed to and subsist on substantially more nutritious elements (White, 2000). They likely process greater fluxes of matter-energy and have bigger budgets than Archaea. This is illustrated in Figure 3 (right) by contracting the Archaeal cloud and stretching the Bacterial cloud along the budgetary axis. In addition, the stressed environments favored by Archaea tend to be fairly stable, such as when stagnant sediments become anoxic, or when still ponds evaporate. Archaea tend to be largely absent from the more variable environments (we describe the examples and argue this point in detail in Datasheet S2 in Supplementary Material). Thus, Archaea appear to have sparser scope and are exposed to fewer signals and less information than Bacteria. Consequently, they are likely to evolve fewer flexibility mechanisms than Bacteria. In contrast, many bacteria are mixotrophic, able to switch between nutrients depending on their availability in the environment (Oren, 2006). These Bacteria are flexible with respect to nutrient kinds and availability. For example, *Rhodopseudomonas palustris* can use thiosulfate, hydrogen gas, sulfur compounds, and possibly CO and formate as electron donors in respiration (Larimer et al., 2004). *Rhodopseudomonas* sp. can use lactate, lamate, butyrate, or acetate as sources of carbon (Barbosa et al., 2001). *Allochromatium vinosum* is able to use hydrogen, sulfide, thiosulfate, sulfur, and sulfite as electron donors, and formate, propionate, furamate, succinate, malate, and glyconate as sources of carbon (Kumar et al., 2008). These Bacteria must process the information associated with the changing nutrient content of the environment. We found no examples of such metabolic flexibility (and metabolic information processing) among archaeons. Table 2 gives examples of archaeal and bacterial species for each strictotrophic and a number of mixotrophic categories. It demonstrates the greater metabolic diversity of the bacterial kingdom as a whole, compared to Archaea. This was illustrated in Figure 3 (right) by rotating and stretching the bacterial cloud further along the information flux axis, compared to the archaeal cloud. Thus, we separated Bacteria and Archaea within their budgetary stratum.

**Table 2:** **Nutritional categories of Archaea and Bacteria**.

	Auto-trophic				Hetero-trophic						
Carbon source	CO_2_				Organic			
Energy for Δp+	Photo-trophic		Chemo-trophic		Photo-trophic		Trophicchemo-		Example Archaea	Example Bacteria	Reference
Source of e−	litho-trophic	organo-trophic	litho-trophic	organo-trophic	litho-trophic	organo-trophic	litho-trophic	organo-trophic			
**STRICTOTROPHS**											
Photolitho-autotroph	X								–	*Chlorothrix* (likely mixotrophic)	White (2000)
Photoorgano-autotroph		X							–	–	
Chemolitho-autotroph			X						Methanobacteria	*Nitrobacter*	White (2000)
Chemorgano-autotroph				X					–	–	
Photolitho-heterotroph					X				–	*Allochromatium warmingii* (likely mixotrophic)	Bergey’s manual of bacteriology
Photoorgano-heterotroph						X			–	Heliobacteria	White (2000)
Chemolitho-heterotroph							X		–	*Silicibacter pomeroyi*	Moran et al. (2004)
Chemorgano-heterotroph								X	*Pyrococcus*	Lactobacillus acidophilus	
**MIXOTROPHS**											
	X				X					*Allochromatium* renukae	Kumar et al. (2008)
			X					X	*Thermoproteus tenax*	Ologitropha carboxidovorans, *Bradyrhizobium*	Paul et al. (2008), Bergey’s manual of bacteriology, Siebers et al. (1997)
			X				X		*Thermoproteus neutrophilus*		Schäfer et al. (1986)
						X		X	Halobacteria		White (2000)
	X					X				*Allochromatium vinosum*	Prange et al. (2004)
	X							X		*Erythrobacter*	Koblízek et al. (2003), Bockstahler and Coats (1993)
	X					X		X		*Rhodospirillum rubrum*	Bergey’s manual of bacteriology
	X				X	X				*Allochromatium* phaeobacterium	Srinivas et al. (2009)
	X				X			X		*Chloroflexus*	White (2000)
	X		X			X		X		*Rhodobacter*	

*The header of this table breaks down the known nutritional categories by the source of carbon and energy. We searched the literature for example Archaea and Bacteria in every strictotrophic category, and as many mixotrophic categories as possible*.

Protista are substantially more flexible than Akarya. In addition to manipulating chemical stimuli, they can process gradients of light, while Akarya can only tell light from dark (Sackett et al., 1997). Metabolic flexibility in Protista is limited to photoorganotrophy. However, they make up for it with other diverse behaviors. Complex, coordinated ciliary, and flagellar beating, modulated by a number of chemical and light stimuli, yields steered locomotion (Jahn and Votta, 1972; Laybourn-Parry, 1984). Many protists, both free-living and parasitic, have complex lifecycles, where each step has its own scope, and the transitions between them can be regulated by a number of environmental signals (e.g., in *Plasmodium*, *Dictyostellium*, *Trypanosoma*, and diatoms). We illustrate Protista as a cloud that overlaps with Akarya but stretches noticeably along the information flux axis (Figure 3, right).

Separating fungi from plants was complicated, as their environmental niches largely overlap. However, we noticed that these niches appeared different through the scope window of the organisms themselves. Specifically, scope density is greater for Fungi than Plants due to the nature of their nutrient sources. Plants use light – a periodically available nutrient. If a plant can survive through the night, nutrients will be available again in the morning. Fungi are saprotrophs and generally consume dead organic matter, which can be in fairly steady supply under dense vegetation due to regular shedding of leaves. Outside of that, dead organisms are not renewable sources of nutrients. The food for Fungi is therefore more ephemeral than food for plants, and therefore it contributes more information to the fungal scope window (Boddy, 1999). Soil fungi constantly explore their environment by degrading starved hyphae and moving the material into those that are actively growing. Thereby the mycelium essentially relocates away from unproductive habitats (Bessey, 1950; Alexopoulos and Mims, 1979; Pollack et al., 2008). This is a complex behavior driven by a number of signals from soil and within the hyphae. It is evidence of flexibility. Plant roots also move through the soil in search of better substrate. However, “whole organism relocation” does not seem to be their innate feature. We illustrate these differences by stretching the fungal cloud along the information flux axis, similar to the case of Archaea-Bacteria separation (Figure 3, right).

Metazoa as a kingdom have the widest spatial dimension of scope, which for some species encompasses spatial variations on a tremendous scale. Traversing environmental gradients, frequently at high speeds (Figure 2) is the trademark of metazoan lifestyle, which endows them with rich, dense scope content. They shuttle through steep thermal and chemical gradients across the landscapes of oceanic and continental vents, sometimes invading areas dangerously close to their thermal death point (Brues, 1927; Mason, 1939; VanDover et al., 2002; Kelley et al., 2005; Tarasov et al., 2005). They traverse wide ranges of thermal, aerobic, and pressure gradients during diving and vertical migration in the ocean (Carey and Scharold, 1990; Takami et al., 1997; Hooker and Baird, 1999; Smith and Brown, 2002; Pearre, 2003; Rex et al., 2006; Jamieson et al., 2009). They process much of this information using sophisticated multicellular sensory organs. Organisms throughout almost the entire Metazoan clade process visual, tactile, auditory, chemical, olfactory, and gravitational signals (Dusenbery, 1992). Clearly, the diversity among metazoan species is tremendous. Most will agree that earthworms probably process fewer signals than lions, and exhibit fewer behaviors. However, the extent to which flexibility has evolved in this kingdom trumps all others. Man transcended the capabilities of his own body by using tools and devices that allow him to explore the deepest trenches of the sea, climb the tallest mountains, and fly through the air and into space! We illustrated this propensity for evolving flexibility by stretching the metazoan cloud far across the information flux axis (Figure 3, right).

What makes metazoan information processing particularly interesting is that they make heavy use of the correlation between different physical signals that are generated by the same source. For example, ground surface temperature and illuminance are correlated. Temperature and pH in the same location of a geothermal pond are correlated. This makes it possible to use one signal (a “proxy”) to make predictions about another, enabling the flexible Metazoa to clamp some of the “important” signals (like temperature) in their optimum range, by using proxy signals to generate the necessary responses. For example, migratory animals cross a wide range of latitudes, and could potentially be exposed to a wide range of temperatures, as expected from the local seasonal variations. However, those animals do not wait for the temperature variations to arrive before they migrate away from the affected area. They use proxy signals, such as the changes in diurnal illumination patterns, to detect the imminent arrival of the climatic change, and move out before it happens. These organisms evolve to be exposed only to a fraction of all temperatures that can potentially occur within their scope size. The use of proxies is facilitated by the large size of metazoan bodies, which literally serve as spatial projection palettes for the diverse patterns of proxy signals, thereby enabling a larger fraction of umwelt in the scope.

### Patterns of robustness

Comparing organisms in terms of robustness is more difficult than comparing them in terms of flexibility. A mechanism of flexibility is frequently readily observable, because it works by incurring change within the organism. In contrast, robustness against a signal is expressed without a response. Consequently, metrics are needed that could serve as reasonable indicators of robustness. Two such metrics seem obvious. One metric is resistance to damage of internal parts and processes. For example, thicker bones are less vulnerable against physical damage than thinner bones. The other metric is the redundancy of internal parts and processes. An organism can carry on after damage to some of its components, if redundant copies are available to take over their function.

It is easy to compare the six kingdoms based on redundancy, including nutrient storage, genetic redundancy, redundancy of microscopic cellular parts, and macroscopic body parts. Storage of nutrients increases robustness of organisms against nutrient shortages. It is used by organisms in all kingdoms but is prominent in most Plants and Fungi, whose vacuoles and specialized tissues serve as containers for a number of useful nutrient substrates (Klionsky et al., 1990; Courties et al., 1994; Nordoy et al., 1995; Marty, 1999; Misumi et al., 2005; Lecointre and Le Guyader, 2006). This is essential to Plants and Fungi; they are sessile and unable to actively search for food. However, Plants and Fungi also use vacuoles for other functions: plants to increase robustness, fungi to increase flexibility. Plant vacuoles enhance robustness against damages by ultraviolet light, by absorbing it with the vacuolar solutes. In mycelial fungi, an extensive system of vacuoles connects hyphae across the mycelia (Veses et al., 2008), and can be used to transport stored nutrients from the satiated hyphae to those transiently in need of nutrients (Bessey, 1950; Alexopoulos and Mims, 1979). When a fungal colony is starved, the vacuoles swell with products of decomposition of the older part of the mycelium, which then are transported to the apical tip to enable growth (Pollack et al., 2008). This flexible use of vacuoles in fungi reflects the disturbed nature of fungal nutrient sources, as well as fungal propensity to “forage” underground.

Genetic redundancy, such as polyploidy, genomic repeats, and multiple gene copies, helps organisms withstand the effects of detrimental mutations. While a few gene copies may be damaged, other copies will remain functional, making the organism robust against mutation (Comai, 2005). Polyploidy is very prominent in plants (estimated 30–80% species, Otto and Whitton, 2000) and multinucleate fungi. However, again its use in the two kingdoms is different. In multinucleated fungi, one hyphal cell can harbor up to hundreds of nuclei (Alexopoulos and Mims, 1979). These nuclei can independently divide, mutate, and move between hyphae through perforations in the septa. Fungal nuclei can even move between two genetically distinct mycelia through hyphal anastomoses, transporting genetic material to new locations and locally generating new phenotypes (Gladfelter, 2006; Croll et al., 2009). Thus, in addition to the robustness benefits, fungal polyploidy promotes genetic flexibility. Genetic redundancy is less prominent in Metazoa than Plants and Fungi, and even less so in Akarya and Protista. Interestingly, in Bacteria the genes present in high copy numbers are usually the highly expressed genes that help increase the throughput of matter-energy and information processing channels by providing multiple copies of the internal subsystems that can work in parallel (Freeman et al., 2006; Popesco et al., 2006). Thus, individuals can match their speed of response to the rate of signal change within a dense scope, or amplify their productivity when nutrients are highly abundant. In other words, high gene copy numbers can make an individual robust with respect to the rate of scope signal change.

Finally, component part redundancy improves robustness against damage to those parts. The only component part redundancy available to unicells is the use of multiple copies of proteins or protein complexes. Multicellular organisms can build organs out of redundant cells, or build redundant body parts (e.g., limbs). The greater is the specialization of cell types and the less reversible the differentiation, the less robustness is imparted onto the individual by the redundancy of cells or organismal parts. Such is the case of Metazoa, where only the lowest forms (e.g., *Hydra*, worms) can continue functioning after parts of their body have been destroyed (Randolph, 1897; Morgan, 1901). In the wild, loss of even a single limb by a higher animal is usually fatal, despite some ability for organ regeneration (Yannas, 2001). Fungi and Plants, on the contrary, stand out due to their redundant and relatively independent leaves, branches (Gill et al., 1995), hyphae (Alexopoulos and Mims, 1979), and even genetically identical ramets within a genet. Plant and fungal individuals are robust to the loss of a substantial number of these “limbs.” Regrowth, as a rule, occurs after loss of a branch or a hypha, and is a flexibility mechanism.

These observations point to a pattern: Fungi and Plants are more robust to damage compared to organisms from other kingdoms, at least based on the metric of redundancy. Fungi tend to use the same redundant feature in a more flexible way than Plants, consistent with our previous observations. Elsewhere in this section we established that Metazoa are distinctly more flexible than the other kingdoms, while unicellular organisms have smallest budget – are most economical.

## The Persistence Strategy Hypothesis

The data presented in the previous section indicate that organisms with a particular propensity toward low budget (greater economy), flexibility, or robustness tend to segregate based on the sizes of matter-energy and information fluxes they process. As a result, the organism clouds in Figure 3 (right) form a triangular shape with vertices corresponding to microbes (economy), Plants/Fungi (robustness), and Metazoa (flexibility). This segregation motivates the hypothesis that tradeoffs operate between economy, robustness, and flexibility. One such tradeoff is between mechanisms by which organisms control the balance between the rates of death and birth in a population. This balance is critical for persistence of a lineage in the context of competition within a variable and potentially hazardous environment (Begon et al., 2006). Flexibility and robustness help decrease death rates by means of withstanding, adapting, or avoiding adversarial environments, and locating beneficial ones. On the other hand, these strategies also cost matter and energy, which otherwise could be spent toward growth and reproduction. Consequently, greater flexibility and robustness result in lower birth rates. So long as births and deaths are in balance, the lineage persists. Less flexibility and/or robustness frees up matter-energy to achieve faster reproduction. This indeed becomes necessary, since the environmental disturbance now is less predictable, less avoidable, and less endurable by individuals, which as a result die at a greater rate. Thus, together flexibility and robustness are in a tradeoff with economy. An organism’s evolved balance among economy, flexibility, and robustness reflects its particular method of persistence, and is of fundamental importance. It corresponds to a particular location on the triangle of organisms in Figure 3 (right), which we therefore call the “persistence triangle.”

Flexibility and robustness are also in a tradeoff relationship with one another, because they compete for matter, energy, and space within the organism. Matter-energy is required to power the parts and the processes that bring forth flexible responses. Yet it is also needed to manufacture and maintain features that confer robustness, such as the redundant internal parts. For this reason, an evolutionary increase in flexibility may lead to sacrifices in robustness, if the budget is maintained, and vice versa. In the next section, we explore which molecular features are correlated with flexibility, in the effort to corroborate this tradeoff.

## Molecular Architecture and Flexibility

Flexibility is measured as the number of responses to external signals, which is likely to increase with the number of different internal processes possible within the organism. In turn, the number of internal processes likely increases with the diversity of the organism’s basic internal components (e.g., cells, molecules, cellular, and molecular parts) and the number of combinations (e.g., multi-part molecules, multi-molecular complexes, networks) that can be put together out of those components. One molecular estimate of internal part diversity is the number of distinct fold superfamilies (FSFs) encoded in the organism’s genome (Caetano-Anollés et al., 2009; Mittenthal et al., 2012). FSFs are groups of families of protein domains that have similar three-dimensional structures and molecular functions, though they may have low identities at the level of protein sequences (Murzin et al., 1995; Chothia and Gough, 2009). The number of FSF is finite and is not expected to exceed much more than ∼2,000 (Levitt, 2009). Statistics of FSFs in genomes are indicative of evolutionary and physiological tendencies of organisms (Caetano-Anollés and Caetano-Anollés, 2005). Thus, FSFs can be viewed as low-level protein building blocks for physiology.

**Figure 4:**
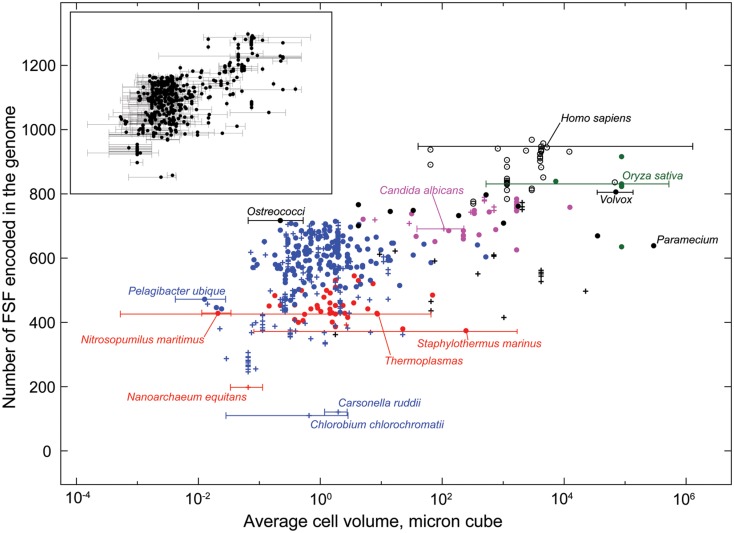
**Average cell volume and diversity of the fold superfamily (FSF) repertoire in 584 fully sequenced organisms**. The number of FSF encoded in an organism’s genome puts a lower bound on the diversity of its internal building blocks. It is thus a measure of the organism’s internal scope size and the potential for information processing and flexibility. An organism’s cell volume is a measure of capacity to contain the processing machinery. This graph shows that, while Archaea and Bacteria have comparable cell sizes, Archaea are more constrained in terms of their building blocks. Most protistan cells are much bigger than akarya, and yet their FSF diversity is comparable. Metazoa have the greatest FSF diversity. Therefore their ability to process information is least constrained by their building blocks. The inset shows ranges of cell volumes for which trusted data could be found in the literature (see Methods). The free-living organisms from the six kingdoms are labeled: red circles – Archaea, blue circles – Bacteria, black filled circles – Protista, magenta circles – Fungi, green circles – Plants, black empty circles – Metazoa. Parasitic and obligate parasitic organisms in the kingdoms of Archaea, Bacteria, Protista, and Fungi are labeled with pluses and the color of their respective kingdom. Key organisms were also marked with cell volume ranges on the main graph. They represent the boundary cases for each kingdom, such as the minimum and maximum cell volume, and the minimum and maximum FSF counts. *Candida albicans* and *Oryza sativa* are not the extreme cases, but are shown for reference. *Nitrosopumilus maritimus* was labeled because it seems to be smaller than the smallest Archaea *Thermoplasma*, but in fact is not. See Datasheet S4 in Supplementary Material for Methods.

**Figure 5:**
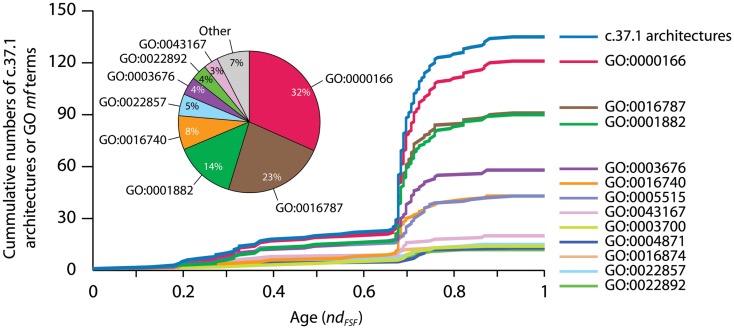
**Accumulation of GO terms of molecular functions (*mf*) associated with the P-loop hydrolase FSF (c.37.1), the most ancient protein domain, and its domain combinations in the timeline of protein evolution**. Wang and Caetano-Anollés (2009) studied the combination of domains in proteins at the level of folds, groups of FSFs that have similar topology, and at the level FSFs that we describe in the figure. A census of protein domain structure and organization at these levels in hundreds of organism was used to reconstruct history using phylogenetic methods widely used in morphometrics. Timelines of protein evolutionary discovery uncovered remarkable patterns, including the explosive appearance of domain combinations during the rise of organismal lineages and the dominance of domain fusion as a pervasive evolutionary force for the generation of protein diversity. Making use of the Gene Ontology Annotation (GOA) system (GOA, 2011), we linked GO terms corresponding to proteins in the UniProtKB database and International Protein Index to sequences with structural assignments. This makes explicit how molecular functions increase as domains combine in protein evolution. The top curve shows the accumulation of c.37.1 FSFs in the evolutionary timeline spanning the origin of proteins (nd_FSF_ = 0) to the present (nd_FSF_ = 1) while the other curves show accumulation of associated GO terms. Time was measured using the nd phylogenetic descriptor (Wang and Caetano-Anollés, 2009). The increase is especially explosive during the “big bang” of domain combinations that occurred more than half way in the evolution of the protein world at nd_FSF_ ∼0.67, a time that coincides with the appearance of FSFs unique to Eukarya (Wang and Caetano-Anollés, 2009). The pie chart shows the abundance of GO-annotated sequences (%) with the c.37.1 FSF in the UniProt database. The three most abundant GO terms (GO:0000166, GO:0016787, and GO:0001882) were also widely distributed in c.37.1 architectures. While the popularity of variants of only six GO terms (among a total of 43 GO term annotations in the UniProt database) was considerably increased by the combination of the c.37.1 FSF domains, the three most abundant GO terms (see pie chart) were also distributed most widely (plots) in the set of functionally versatile architectural variants of the c.37.1 FSF. Thus, each new instance of an FSF domain in a proteome implies its use within a different molecular and cellular context, often in molecular functions that are more structurally and evolutionarily derived than those originally intended for the structure. GO:0000166, nucleotide binding; GO:0016787, hydrolase activity; GO:0001882, nucleoside binding; GO:0003676, nucleic acid binding; GO:0016740, transferase activity; GO:0005515, protein binding. See Datasheet S4 in Supplementary Material for Methods.

**Figure 6:**
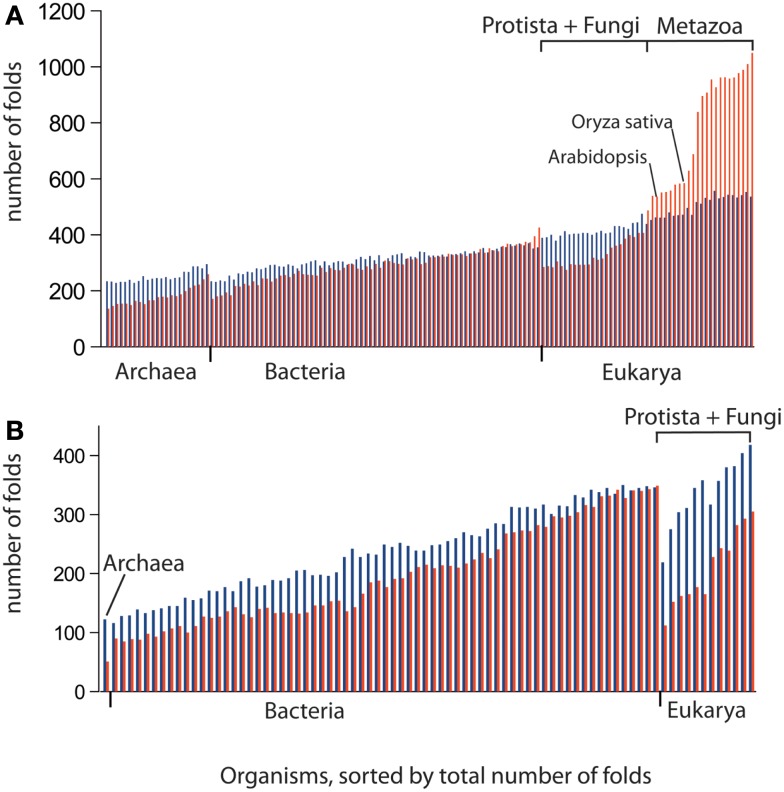
**The combinatorial use of protein architectures correlates with propensity for flexibility**. The number of distinct folds, which tend to participate only in single-domain proteins (blue bars) and in multi-domain proteins (combinatorial folds, red bars), is displayed in the order of increased diversity of folds in genomes. The greater use of combinatorial than single-domain folds in Metazoa is consistent with their propensity for flexibility rather than robustness, in comparison with plants and fungi. Bacteria use combinatorial folds to a greater extent than Archaea. This is also consistent with our prediction that Bacteria is more flexible than Archaea. **(A)** Free-living organisms. **(B)** Obligate parasites. See Datasheet S4 in Supplementary Material for Methods.

**Figure 7:**
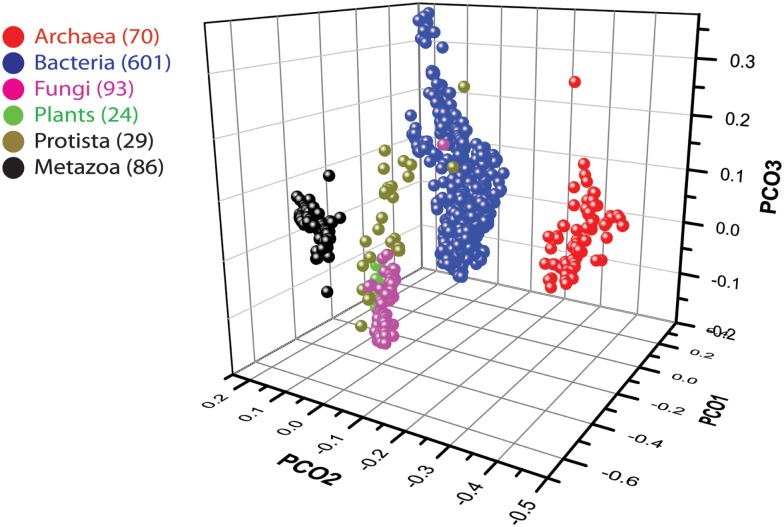
**Plot of the first three axes of the PCoA describing similarities between the proteomes of 903 organisms based on the Pearson correlation similarity matrix**. See Datasheet S4 in Supplementary Material for Methods.

We used data from fully sequenced organisms to evaluate whether FSF diversity is a good correlate of flexibility. We plotted the total number of distinct FSFs against characteristic cell volume in Figure 4 (see also Datasheet S3 in Supplementary Material). Cell volume correlates with the cellular capacity to contain the machinery for processing matter-energy and information and is indicative of cellular budget. The resultant grouping of organisms turned out to be very similar to that in Figure 3 (right), suggesting that FSF diversity can serve as an approximation of an organism’s information flux. However, the number of FSFs is limited, and that constrains the complexity of an organism’s physiology built with single-domain proteins alone, even though the number of possible protein structures is very high (Andreeva and Murzin, 2010). Use of multi-domain proteins dramatically expands the diversity of protein organization and the diversity of associated biological functions (Bashton and Chothia, 2007). Each new instance of an FSF domain in a proteome implies its use within a different molecular and cellular context. Often the domain is used in molecular functions that are more structurally and evolutionarily derived than those originally intended for it. Figure 5 illustrates this fact with a structural and functional analysis of the P-loop hydrolase FSF, the most ancient domain structure in the protein world. Can the number of domain combinations serve as a better correlate of flexibility? Wang and Caetano-Anollés (2009) studied the combination of domains in proteins at the level of FSFs and folds (groups of FSFs that have similar topology). They showed that some folds participate only in single-domain proteins (single-domain folds) and some only in multi-domain proteins (combinatorial folds). We made use of their published data to show that organismal kingdoms have different preferences for the balance between using single-domain and combinatorial folds for free-living species (Figure 6). The data show that organisms from the more flexible kingdoms tend to have more combinatorial folds than single-domain folds. This is the case for all Metazoa that have been fully sequenced by 2008 (22 animals). Some animals use up to twice as many combinatorial folds than single-domain folds. In contrast, each of the 43 species of Archaea has noticeably more single-domain folds than combinatorial folds. Bacterial species appear to keep approximately equal representation between the two kinds of folds, which is consistent with their expected higher flexibility, compared to Archaea. The distinction within the Fungi-Plant stratum is less clear. However, the *distributions* of FSFs in proteomes finally dissected the flexibility contributions in the six organismal kingdoms. We performed multivariate statistics of a presence/absence matrix for all FSF in the fully sequenced proteomes. A Principal Coordinate Analysis (PCoA) separated the organisms into the corresponding six kingdoms with minimal data manipulation when similarities were measured in terms of Pearson’s correlation coefficients (Figure 7) or Euclidean distances (data not shown). The relative position of the organism clouds in the PCoA three-dimensional plot is consistent with all the other patterns we mentioned in the preceding sections. These results strongly suggest that every kingdom evolved its own strategy for utilizing the diversity of the available protein architectures, and the distributions of FSFs are an adequate metric of flexibility.

## Molecular Architecture and Robustness

Before leaving the subject of protein folds, we comment on how they can help us quantify robustness. Robustness can be conferred to an organism when its building blocks are less vulnerable to a signal. For example, an organism that inhabits an extreme environment is more robust if its proteins are less subject to denaturation in those extreme conditions (Kumar and Nussinov, 2001; Berezovsky and Shakhnovich, 2005; Zeldovich et al., 2007). The need for protein structures that are less vulnerable is especially great for microbes in thermophilic conditions. Single cells have few means to control their internal temperature. Unicellular organisms must repair the damage incurred by extreme temperature, and evolve protein structures resistant to the damage. We hypothesize that temperature limits the number of viable protein architectures. Organisms adapted to thermophilic conditions are expected to have fewer distinct FSF in their genomes, compared to mesophiles. Mesophiles do not experience the constraints of thermophily, and are free to evolve more kinds of FSFs, many of which are not robust with respect to temperature. Thus, mesophiles have greater evolutionary potential for flexibility, due to the lack of evolutionary pressure against FSF diversity. Thermophiles, in contrast, are under evolutionary pressure to evolve robustness through damage-resistant FSFs, and their flexibility is constrained due to lower FSF diversity. We collected the data on FSF diversity and environmental parameters (Datasheet S3 in Supplementary Material) to test this hypothesis in the case of free-living akaryotic microbes. Indeed, the average numbers of distinct FSFs present in mesophilic organisms were found significantly greater compared to thermophiles, within each akaryal kingdom (Table 3). The average number of distinct FSFs present in Archaea was significantly smaller compared to Bacteria, both for mesophiles and thermophiles. This result suggests that regardless of thermophilic constraints, Archaea at large tend to have smaller FSF diversity than Bacteria. Smaller FSF diversity is thus a property of the kingdom, indicating that Archaea have evolved (perhaps from a hyperthermophilic ancestor; Gribaldo and Brochier-Armanet, 2006) within a different persistence strategy (trading flexibility for robustness) than Bacteria (trading robustness for flexibility).

**Table 3:** **Thermophily restricts FSF diversity**.

Mesophilic Archaea	*p* < 0.001, d*f* = 41	Thermophilic Archaea
Mean = 478.0		Mean = 428.5
STD = 17		STD = 27.3
*N* = 43.2		*N* = 26
*p* < 0.001, d*f* = 198		*p* < 0.001, d*f* = 47
**Mesophilic Bacteria**	*p* < 0.05, d*f* = 204	**Thermophilic Bacteria**
Mean = 601.5		Mean = 554.5
STD = 75.6		STD = 41.3
*N* = 183		*N* = 23

*Two-tailed unpaired t test performed (with Bonferroni correction) on the FSF diversity in mesophilic and thermophilic members of Archaea and Bacteria that are not obligately parasitic. All pairwise comparisons of mean FSF diversity between the four classes of organisms yielded statistically significant results*.

**Figure 8:**
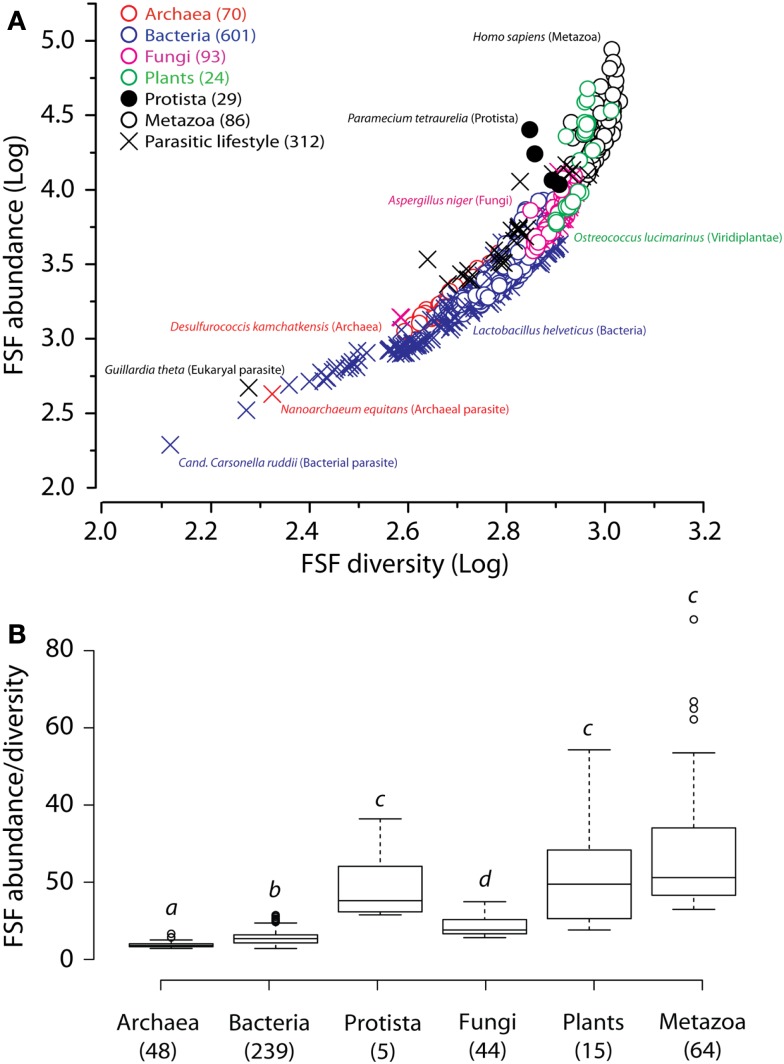
**Plots of FSF use (diversity) versus FSF reuse (abundance)**. **(A)** Plot of FSF use and reuse in the proteomes of the entire set of 903 organisms. **(B)** Box plots of the ratios of FSF reuse to FSF use in the proteomes of 415 free-living organisms of the six kingdoms. The ratio of 1 would imply that every FSF is found exactly once in the entire genome, which is why all numbers on the ordinate are greater than 1. Numbers in parenthesis indicate total number of proteomes studied for each group. Horizontal lines within each distribution indicate group median values. Outliers are indicated by hollow circles for Archaea [*Methanospirillum hungatei* (ratio = 5.7), *Methanosarcina barkeri* (5.7), *Methanosarcina acetivorans* (6.7)], Bacteria [*Saccharopolyspora erythaea* (10.0), *Streptomyces griseus* (10.0), *Streptomyces avermitilis* (10.2), *Streptomyces coelicolor* (10.3), *Solibacter urisatus* (10.6), *Rhodoccus* sp. (11.0), *Burkholderia xenovorans* (11.1), and *Sorangium cellulosum* (11.5)], and Metazoa [*Homo sapiens* (62.2), *Monodelphis domestica* (65.0), *Branchiostoma floridae* (66.9), *Takifugu rubripes* (88.1)]. Raw data was transformed by its reciprocal to meet the assumption of normality for one-way ANOVA. Welch’s correction was applied to protect from heteroscedastic variances (Welch, 1938) and Games–Howell multiple comparison test (Games and Howell, 1976) was used to evaluate significant differences among individual groups at *P* < 0.02 (indicated by different letter heading box plots).

Multiple occurrences of a protein domain within a genome are a form of redundancy. Those may correspond to proteins with domain repeats or straight gene redundancy described above. We plotted the total number of all FSF domains (reuse) to the number of distinct FSFs (use) for the genomes of the 903 organisms described above (Figure 8A). Any departure from a straight-line indicates an increase in the use and reuse of FSF domains and serves as a metric of robustness. Since evolutionary reductive lifestyles within kingdoms can bias general trends (Wang et al., 2007), we excluded parasites and symbionts and focused on 415 free-living organisms. Remarkably, we find on average a higher FSF reuse-to-use ratio in Bacteria compared to Archaea, and in Plants compared to Fungi (statistical *p*-value cutoff at 0.02; Figure 8B). Combined with the above considerations of thermophily and redundancy, these data suggest that plants increase their robustness by increasing redundancy of their internal parts, starting at the genomic and proteomic level, and up to the whole body level. On the other hand, Archaea have not evolved significant genomic and proteomic redundancy. These data are in agreement with previous observations that Archaea underwent significant reductive evolution after the split from the last universal common ancestor (LUCA) of cellular life (Wang et al., 2007). Their robustness therefore arises from lesser vulnerability of the parts themselves.

## Evolutionary Dynamics of the Framework

**Figure 9:**
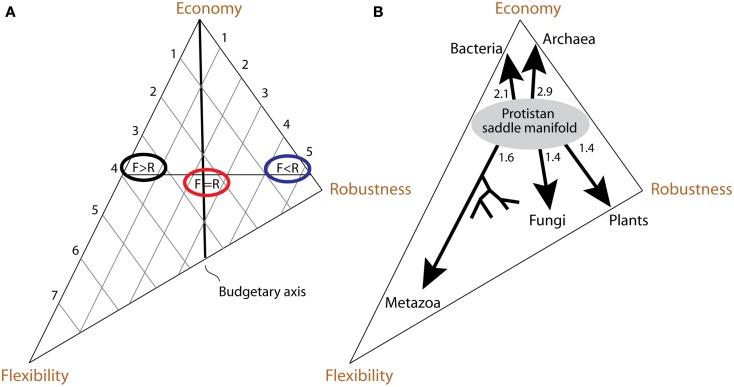
**Interpretation of the persistence triangle and evolution within it**. **(A)** If flexibility and robustness could be measured quantitatively, then its construction and interpretation would be fairly similar to Grime’s triangle of plant life strategies (Grime, 1974). The flexibility and robustness measures would be plotted along their respective axes, and organisms could be characterized and compared based on their placement within the triangle. For example, the organism with flexibility 4 and robustness 1 would be placed fairly close to the flexibility axis (black ellipse). The organism with flexibility 1 and robustness 5 would, on the contrary, be positioned closer to the robustness axis (blue ellipse). Somewhere in-between them is a region where organisms are equally flexible and robust (red ellipse). By definition, such organisms are located on the “budgetary axis.” **(B)** Evolutionary paths within the persistence triangle take the form of branching patterns, going either mostly toward the economy vertex (reductive evolution), or away from it (expansive evolution). We propose the differences in nature and evolution of organisms result from their tendencies to evolve toward different tradeoff solutions on the persistence triangle. Archaea and Bacteria undergo reductive evolution relative to Eukarya, with Archaea evolving robustness and Bacteria flexibility. Within Eukarya, there are three economic strata. The first one is occupied by Protista, many of which ride the viscosity barrier. Other eukarya are above the viscosity barrier and evolve expansively. In the second economic stratum Fungi evolve flexibility and Plants evolve robustness. In the third stratum Metazoa evolve flexibility, skewing the triangle in that direction. Approximate times of origin of kingdom expansions from the ancestral protistan manifold are indicated in billion of years and are based on the molecular clock of protein architecture (Wang et al., 2011).

Our primary purpose for introducing the economy/flexibility/robustness trio was to develop a method of representing organisms in a comparable way. The resultant persistence triangle allows us to place any organism and relate its persistence strategy to that of other organisms, regardless of the differences in their levels of complexity or environmental niche. The persistence triangle is a model, which formalizes the properties and coevolution of the two trios – scope/umwelt/gap and economy/flexibility/robustness (Figure 9A). This section explores those properties and coevolution.

### Coevolution of the two trios

Because scope content, and its division into umwelt and gap, depend on the way individuals explore their environment, it is fair to presume that the elements of the scope/umwelt/gap trio coevolve. At birth an individual is endowed with a certain set of evolved sensory organs and behaviors (and whatever unique mutations it might have) that predispose it to having a particular scope content. The individual samples the set of possible signals over the course of its lifetime, populating its umwelt and gap. The individual’s unique experience due to its own mutations, unique behavior, or local changes in the environment stemming from natural geological, climatic, and other variations, deviates from the scope that might have been expected at birth. As the genotypes of individuals evolve, their interaction with the environment changes. So do the scope content and its division into umwelt and gap. Let us consider, for example, the evolution of proxies. Animals avoid exposure to forest fires by using olfactory (combinations of chemical signals), visual (patterns of illumination wavelengths and intensity), and auditory (pressure variations) cues. The more flexible organisms evolve more sophisticated sensors (e.g., eye) as well as behaviors (e.g., sniffing), to extract more information from the proxy signals. As flexibility mechanisms evolve, some of the scope signals that are outside the organism’s optimum physiological range can become replaced with their proxies. This enriches the umwelt with the patterns of changes and combinations of different proxy signals, and shrinks the gap.

The correspondence between the scope/umwelt/gap and economy/flexibility/robustness is dynamic, not static. They coevolve together. Larger, more mobile organisms that have bigger scope are exposed to more signals. Such organisms are under greater selective pressure to evolve mechanisms of flexibility and robustness. Simultaneously, having more diverse mechanisms of flexibility and robustness should make it possible for organisms to venture into new territories, and be exposed to even more signals. This can result in progressively expansive evolution, which trades economy for flexibility, and robustness. Because flexibility and robustness mechanisms compete for matter-energy, organisms are expected to evolutionarily branch out into lineages that trade one for another within this expansive trend (Figure 9B). The branching is also expected within the process of reductive evolution. Organisms with smaller scope are also exposed to fewer signals, which reduce the selective pressure to evolve mechanisms of flexibility and robustness. Those strategies are evolutionarily traded for economy. This can result in progressively reductive evolution, to enable higher reproductive rates required to persist under the influence of now less predictable and less avoidable (though more rare) environmental signals. Within the same small budget, however, organisms will still branch out to evolve mechanism of flexibility and robustness with respect to some of the few signals to which they do get exposed. Does the persistence triangle have a boundary between the initial states that predispose toward expansive and reductive evolution?

We hypothesize that protist-like unicellular organisms form a kind of saddle manifold in the persistence triangle (Figure 9B), and thus represent such boundary. Organisms tend to evolve reductively on the economy side of this manifold, and expansively on the opposite side. We believe that viscosity of water historically set up the manifold. Viscosity sets the critical organism size (viscosity barrier) at which motility becomes useful for nutrient acquisition: about 100 μm. Below that size, there is negative selection pressure on motility speed due to its high energetic cost. This pressure results in small scope size and a lack of positive selection for the mechanisms of flexibility and robustness. These organisms can neither predict nor escape environmental disturbances. That, plus predation by bigger organisms, puts them on the path of reductive evolution toward the economy vertex (i.e., Akarya). Above the critical organism size, it becomes possible to chase and engulf other organisms. Bigger spatio-temporal range and predatory lifestyle expand and enrich the scope, putting a positive selection pressure on evolution of flexibility and robustness. This sets organisms on the path of expansive evolution with the divergence into those evolving toward enhanced flexibility (i.e., Metazoa) or robustness (i.e., Plants).

The described processes of reductive and expansive evolution along the budgetary axis have been previously discussed in the literature as the trends of evolution within the r/K continuum (e.g., Reznick et al., 2002). R-selected organisms have high growth rate, short lifespan, and produce many offspring with a low survival rate. K-selected organisms operate close to their environment’s carrying capacity. They live longer and produce fewer offspring with higher survival rate due to high parental investment. The economy vertex is a cognate of the r-selected extreme on the once-popular r/K continuum. Our framework adds a new dimension to this continuum: the axis of flexibility/robustness, which turns the r/K continuum into a branching structure (Figure 9B), more congruent with the usual depiction of the tree of life. Our depiction of branching evolutionary pathways within the persistence triangle is based on the function of an individual’s physical components and parts, as opposed to their structure, which formed the basis for building most trees of life to-date. The two approaches are complementary. Let us further discuss the molecular aspects of evolution of persistence strategies.

### Molecular constraints on evolutionary movements within the persistence triangle

The persistence triangle embeds two tradeoff relationships. First, economy is in a tradeoff relationship with both flexibility and robustness. Second, flexibility and robustness are in a tradeoff with each other. If flexibility and robustness could be measured directly, we could draw the persistence triangle (Figure 9A) as it has been done for plant life strategies (discussed below; Grime, 1974). This persistence triangle is asymmetric, for three reasons. First, flexibility mechanisms tend to cost more matter-energy than robustness properties. All molecular constituents of an organism must be manufactured and maintained, regardless of their purpose. However, most of the elements conferring robustness just “sit there,” incurring no further cost (e.g., bark, thorns). In contrast, the information processing pathways that confer flexibility with respect to a particular signal use extra matter-energy when they process that signal. This difference in costs means that evolving an additional flexibility mechanism can expand budget more than evolving an additional robustness mechanism from the same location on the triangle. Consequently, the flexibility edge of the triangle is longer than the robustness edge (Figures 3 and 9). Second, the space of possibilities for evolving flexibility is greater than that for robustness. Flexibility is correlated with the diversity of an organism’s building blocks. Organisms generate that diversity by evolving new building blocks, and by combining them with each other in various permutations, thereby creating multiple nested levels of complexity. Therefore, each additionally evolved building block creates opportunities to manufacture not one, but many new internal subsystems, potentially conferring flexibility against many different signals. Each of these new flexibility mechanisms can then serve as a starting point for further evolution. In contrast, robustness is correlated with redundancy of building blocks. Each additionally evolved building block that is robust against a particular signal confers robustness to the organism only with respect to that signal alone. Fewer uses for a robust mechanism translate into fewer opportunities for further evolution, compared to flexibility mechanisms. Third, evolution of flexibility begets more flexibility, and evolution of robustness begets more robustness. Flexibility is brought forth through information processing subsystems, e.g., sensors, transduction channels, and deciders. Through mutational modification, sensors are likely to generate more sensors (as, for example, is the case for cellular membrane ion channels; Anderson and Greenberg, 2001; Pohorille et al., 2005). Deciders are likely to generate more deciders, e.g., genetic regulatory elements (Ludwig, 2002) and neural networks (Bullock, 2002). Similarly, the molecular components that confer robustness tend to generate more components that contribute to robustness (e.g., collagens; Exposito et al., 2002). Combined with the greater space of evolutionary possibilities for flexibility, these processes result in the asymmetry of the persistence triangle. The flexibility edge is longer than the robustness edge. We hypothesize that the outlined self-reinforcement of flexibility and robustness results in evolutionary positive feedback loops that funnel lineages preferentially into those strategies. These loops must operate synergistically with the processes of expansive and reductive evolution.

### Gray areas in the persistence triangle

The nice picture of the separating positive feedback loops for flexibility and robustness is somewhat muddied by economic constraints. Indeed, the division between umwelt and gap is exclusive: the same signal can be in either part of the scope, but not in both. However, the dominions of flexibility and robustness over signals overlap. Furthermore, multiple flexibility and robustness mechanisms can exist for the same signal. Here are three examples of managing signals at high cost:

- An organism may have more than one flexibility mechanism with respect to a single signal. This increases the expense of processing that signal. For example, we withdraw a hand from fire, and then blow on it to ease the pain – both responses to the same signal of pain.
- An organism may have more than one mechanism of robustness with respect to the same signal. This increases the expense of exposure to that signal. For example, both thick fur and layer of subcutaneous fat serve to make the arctic fox less vulnerable to cold.
- An organism may have both flexibility and robustness mechanisms with respect to the same signal. For example, fawns are both robust and flexible with respect to proximity to a predator. Coloration makes them difficult to see (robustness), and they freeze in response to the sight of predator (flexibility).

There are also ways of managing signals at lower cost:

- Applying a single blanket mechanism that works against multiple signals can decrease the costs of managing signals. For example, the avoidance behavior in Metazoa (a flexibility mechanism) can be used against the sight of a predator, a noxious smell, or extreme heat. Tree bark (a feature imparting robustness) provides protection against excessive evaporation as well as against mechanical damage. The problem is, a single blanket mechanism may not be optimal in every case it is used. Thus, blanket mechanisms promote economic use of resources while sacrificing survivability.
- A single body part or feature can be used to enhance both flexibility and robustness: two persistence strategies for the price of one body part. For example, a peacock’s tail can be fanned to attract females – a flexible response to the sight of a potential mate (Loyau et al., 2007). Its conspicuous coloration pattern may also serve as anti-predator robustness mechanism (Baker and Parker, 1979).

Finally, there are intermediate mechanisms that are difficult to classify as either flexibility or robustness. For example, repair processes are usually initiated in response to an internal stimulus indicating that damage occurred. On the one hand, repair is a mechanism of flexibility with respect to internal signals of damage. On the other hand, repair contributes to robustness with respect to external signals that are causing the damage. It allows the organism to remain under the influence of that signal without processing it, but withstanding it by constant repair of whatever damage it might have induced. Similarly indeterminate is the case of sporulation in unicells, which is a flexible response to external stimuli of starvation and desiccation, and which induces the state of heightened robustness. Repair and sporulation are also blanket mechanisms. Repair works with respect to any signal that induces damage in the organism. Sporulation works with respect to a wide range of stresses.

These overlaps between flexibility and robustness create gray areas in the persistence triangle. First, because blanket mechanisms work against a number of signals, evolution of one such blanket mechanism can move the organism laterally far away from the economy axis, without much increase in the budget. Second, co-option of a single mechanism into both flexibility and robustness improves the organism’s survivability without much impact on the budget (True and Carroll, 2002). Third, flexible responses can evolve by capitalizing on pre-existing information processing pathways, again preserving budget (e.g., sensory drive Wyatt, 2003). Thus, two organisms may occupy the same location (or at least the same stratum) in the triangle, but have different degrees of flexibility and robustness.

These ways of improving flexibility and robustness without much impact on budget create an evolutionary “room to expand” within any single budgetary (economic) stratum. Exploration of this gray area yields economically comparable organisms with diverse mechanisms of flexibility and robustness, perhaps resultant from adopting inhomogeneities within the same environmental niche. For example, the beak shape in Darwin’s famous Galapagos finches is related to the beak’s robustness with respect to food manipulation. The strength and timing of expression of the bone morphogenic protein four and of calmodulin during bird embryonic development seem to account for the wide diversity of beak shapes (Abzhanov et al., 2004, 2006; Wu et al., 2004). Thus, the diversity in beak robustness results from evolutionary exploration of the expression patterns of only two genes, without much impact on economy of the individuals.

At some point during exploration of this gray area, a lineage may break into the next economic stratum. A substantial loss of flexibility or robustness mechanisms will cause the organism to undergo reductive evolution and enter the economic stratum with lower budget. Invention of a new, more costly mechanism of flexibility or robustness causes the organism to undergo expansive evolution and enter the economic stratum further away from the economy vertex of the persistence triangle. This process is congruent with the theory of punctuated equilibrium (Gould and Eldredge, 1993). The gray areas of lateral evolution on the triangle within the same economic stratum correspond to the periods of “stasis”. The jumps to the adjacent economic stratum, via expansive or reductive evolution that result from a significant change in flexibility/robustness mechanisms correspond to the “punctuated change.”

These hypotheses allow us to propose a model for evolutionary separation of LUCA into the three cellular superkingdoms, as follows.

### Evolutionary segregation of the three superkingdoms

The viscosity-bound protistan manifold separates the modern Akarya from the vast majority of modern Eukarya, and therefore seems to be a reasonable candidate for placement of a primordial organism, before the three superkingdoms split off into diversified forms. This placement would be consistent with an earlier hypothesis that the ancestor of the three superkingdoms was a relatively large (larger than Akarya) phagotrophic organism (Poole et al., 1999; Kurland et al., 2006). However, it says nothing about the timing and order of the diversification process, or the steps of change in cellular make up that led to this primordial entity.

It is likely that phagotrophy placed the selective pressure for reductive evolution on smaller organisms. If that is the case, bacterial ancestors embraced their status as prey. They went on evolving r-selected traits, taking advantage of habitats with abundant nutrients, where they adapted to reproduce quickly and compete ruthlessly for the available resources. The opportunistic and highly social lifestyle promoted evolution of whatever flexibility mechanisms could be managed within their tiny budget. Flexibility also served well in their interactions with predators. Thus, the ancestors of Bacteria split off the primordial saddle manifold, and slid down into the potential well of viscosity-driven, economy-promoting world, with a bias toward the flexibility in that corner of the triangle. The ancestors of Archaea took an alternative reductive path biased toward robustness. They settled in flexibility-constraining thermophilic niches, inaccessible to ancient bacteria and the phagotrophic common ancestor. These ancestral archaeal lineages escaped from both competition and predation. The thermophilic environment constrained their molecular stability, causing them to lose a large number of the primordial proteins (Poole et al., 1998; Wang et al., 2007), locking them on this path of reductive evolution with a bias toward robustness.

The phagotrophic members of the early organisms derived from the primordial cellular ancestor went on to produce the ancient eukaryal lineage, perhaps when some of them managed to assimilate and establish a symbiosis with members of the evolving bacterial lineage (Cavalier-Smith, 2002b; de Duve, 2007; Jékely, 2007). This putative symbiosis could have provided the means of extremely efficient matter-energy extraction from the nutrients necessary to support the costly lifestyle of the budding predator. The new lineage quickly out-competed its less efficient ancestor, and resulted in the primordial protozoan. Remarkably, the proteomic make up of Bacteria and Protista share distribution patterns of molecular functions that suggest an ancestral evolutionary link between these two groups (Nasir et al., 2011). From there evolution proceeded as described before. The ancestors of the remaining eukaryal kingdoms slid down the protistan saddle manifold in the direction of ever-increasing budget, each with a bias toward flexibility or robustness, appropriate for their nutritional lifestyle.

In short, we propose that LUCA split into thermophiles, competitors and predators, which went on to become Archaea, Bacteria, and Eukarya, respectively. Subsequently Archaea moved into other extreme environments, where they could thrive despite the energy-stressed conditions (Valentine, 2007) due to their very small budget and low competition. Through secondary adaptations, some bacteria also moved into the extreme environments, and some archaeaons became mesophiles. Bacteria went on to perfect their flexible but highly reduced phenotype in form of parasites. Secondary adaptations in Protista, Fungi and Metazoa (each gravitating toward flexibility within their own economic stratum) also resulted in emergence of parasitic lineages. The robust plants had minimal opportunities to evolve such forms.

## Summary and Discussion

### The new synthetic evolutionary framework in systems biology

In this work, we aimed to come up with a general, synthetic, and scalable framework that would describe organisms’ methods of persistence. We were motivated by the desire to understand the essence and fundamental differences between very large groups of organisms. We chose kingdoms and superkingdoms. The organisms in those groups are very different and comparisons performed thus far used criteria and language that were idiosyncratic to the particular comparison. We wished to come up with a language that would work for any comparison. To that end we founded our framework on Miller’s theory of living systems (Miller, 1995), which represents all organisms is the same way.

Miller’s theory describes every organism as a network of the 20 internal subsystems, which process matter, energy and information. Some subsystems, such as ingestor, matter-energy storage, and producer, process only matter-energy. Some subsystems, such as input transducer, associator, and memory, process only information. Some, such as reproducer, process both. The theory establishes properties and quantitative descriptors for every subsystem, by describing their structure and process. Finally, it offers a number of predictions about the relationships between subsystems. This theory of living systems was developed by Miller over the course of several decades in collaboration with historians, anthropologists, economists, political scientists, sociologists, psychologists, chemists, physicists, physiologists, and medical doctors. It is very detailed and provides a solid base for any comparative, analytical or synthetic work in systems biology. However, it does not have good descriptors of the organisms’ environments that would work at all spatio-temporal scales. It was thus essential for our purposes to be able to compare the environments characteristic of different organisms and to perform that comparison in the context of persistence.

Life history theories have developed language to describe the environment in the context of lineage persistence. Grime’s universal adaptive strategy theory (UAST) seems particularly attractive, as it uses very general yet intuitive descriptors of the environment, identifying three life strategies: competitive, stressed, and ruderal (Grime, 1974). Grime’s theory was very successful, as it has been supported for organisms within each of the kingdoms (Grime, 1977; Grime and Pierce, 2012). However, it cannot capture persistence methods of the entire kingdoms and superkingdoms. Superficially, it may appear that Bacteria, Archaea, and Eukarya can be elegantly described as competitive, stress-tolerant, and ruderal, respectively. Bacterial competitiveness is notorious, and Archaeal adaptation to energy-stressed environment has been convincingly demonstrated. However, their life histories are very similar, whereas UAST theory predicts separation based on reproductive rate: high for competitors and low for stress-tolerant organisms. Similarly, Eukarya may seem to persist in ruderal environments, which is characterized by high disturbance (e.g., intermittent availability of prey for predatory eukaryotes) and low intensity stress (i.e., due to heterotrophy). However, Eukarya do not seem to fit the profile of ruderal organisms, which are expected to have rapid life cycle and produce large amount of offspring. Some eukaryal lineages fit this profile (e.g., fish and insects compared to mammals; budding yeast compared to perennial plants), but not the entire superkingdom as a group, when compared to Archaea and Bacteria. UAST is based on the premise that organisms apportion their material resources between growth, maintenance, or regeneration – three needs that compete for matter and energy. It does not take into account the use of information, which according to Miller’s theory is crucial for living systems.

We here propose a hybrid framework that derives persistence strategies based on both the use of matter-energy and information. The latter was incorporated through umwelt. Jacob von Uexkull studied the relationship of animals with their environment and emphasized the meaning of environmental signals for animals (von Uexküll, 1909). The semiotic aspect was not necessary for our framework, but we made use of the concept of umwelt because it allowed us to represent the environment from the perspective of the organism. This made the niches of organisms from all kingdoms comparable. The result is the trio of persistence strategies: economy, flexibility, and robustness. Every lineage evolves a unique tradeoff solution among these strategies. These solutions are useful for comparison and classification of organisms and endow our framework with considerable descriptive and explanatory power.

This novel framework integrated data on life histories, morphology, nutrition, and molecular organization. The metabolic theory of ecology (Brown et al., 2004) was instrumental in analyzing the relationship between scope size and budget. The r/K selection theory (Pianka, 1970; Boyce, 1984; Reznick et al., 2002) turned out to be embedded in our framework by way of the organism distribution along the economy axis. The dichotomy between flexibility and robustness resolves between Archaea and Bacteria, and between Plants and Fungi, which could not be done using the r/K selection principles. Through this integration of data and theories, our synthetic model makes an important contribution to systems biology, because our persistence triangle and the evolutionary movements within it depict an important unifying functional and organizational principle that explains the fundamental differences between organisms.

### Persistence triangle is a complete and universal model of persistence strategies

Economy, flexibility, and robustness are both necessary and sufficient to describe the full set of persistence strategies. This trio was derived based on the division of scope into umwelt and gap. The umwelt and gap cover scope completely. Any signal is either processed by an organism, or not. If it is processed, it is part of the umwelt, and a flexibility mechanism is in place to process it. If the signal is not processed, then it is part of the gap, and there is not a flexibility mechanism in place for it. Depending on the budget, there may or may not be a robustness property in place to withstand the effects of that signal. Thus, flexibility and robustness completely describe what an organism does with information it encounters. Economy takes into account competition between organisms for matter/energy. Hence, the economy/flexibility/robustness trio is necessary and sufficient to fully describe an organism’s persistence strategy. Other factors, such as resilience, adaptivity, and vulnerability, have been considered in the literature, but they are either synonymous with or tangential to economy/flexibility/robustness. Unfortunately, the terms robustness, resilience, flexibility, and others, are used differently in mechanical engineering, computer engineering, software design, and biology. One has to follow the definitions when comparing our framework and conclusions to the models that are based on observations in those disciplines.

We believe that our framework is universally applicable to all classes of organisms. It works at the level of kingdoms, can be generalized to superkingdoms, and is able to resolve closely related organisms, such as Darwin’s finches.

### Outlook

Three components interplay within our framework: ecological, molecular and evolutionary. Describing all three satisfactorily would result in a publication of substantial volume, well beyond the confines of this publication. Thus, here we focus on the ecological component and will discuss the other two components elsewhere. However, the present ecologically oriented exposition allows us to pose useful questions to be addressed from the molecular and evolutionary perspective.

A number of observations and suggestions concerning the evolution within the persistence triangle could bear further testing. We made a reasonable conjecture when we discussed molecular constraints on evolutionary movements within the persistence triangle that flexibility on average has greater costs than robustness. The resultant hypothesis is that addition of novel mechanisms of flexibility tends to stretch matter-energy budget more than the addition of novel robustness properties. This could be demonstrated by comparing metabolic rates of closely related organisms that have distinguishable persistence strategies. Miller’s theory can help identify the subsystems responsible for the difference in strategies. It can also help determine whether mechanisms of flexibility are generally more costly than robustness properties. This can be done by computing their costs of manufacture, maintenance and operation for comparable subsystems.

We also suggested that evolving any of the three persistence strategies predisposes the lineage toward further exploitation of that same strategy. Flexibility begets more flexibility, robustness begets more robustness. This sets the stage for known patterns of reductive and expansive evolution along the economy axis. It would be interesting to explore examples of these processes for molecules and pathways. Perhaps there are patterns within each of these evolutionary “positive feedback loops.” Additional constraints on the evolutionary paths within the persistence triangle can be discovered through this exploration.

The diversity and abundance of internal parts of an organism, such as FSF domains, not only dissects organisms into kingdoms (Figures 5–8) but also acts as good repository of organismal history (reviewed in Caetano-Anollés et al., 2009) and can help confirm the evolutionary generalizations we are here deriving from the persistence triangle. Since each new instance of reuse of an FSF is costly and requires genes to duplicate and diversify, genomic abundance is correlated to time. A model in which increases in genome occurrence unfold progressively in evolution of proteomes, coupled to standard cladistic principles (Caetano-Anollés and Caetano-Anollés, 2005; Wang et al., 2007; Kim and Caetano-Anollés, 2011), can formalize and systematize the placement of organisms in the persistence triangle, this time within a phylogenetic framework. The model of protein evolution predicts a molecular clock of FSFs (Wang et al., 2011) that links phylogenetic statements derived from phylogenomic analysis to the geological record (Wang et al., 2011; Kim et al., 2012). Remarkably, analyses of domain structure and organization at fold, FSF and fold family levels suggest a very early origin of Archaea that was reductive, followed by later origins of the lineages of Bacteria and Eukarya, in that order (Wang et al., 2007; Wang and Caetano-Anollés, 2009; Kim and Caetano-Anollés, 2011, 2012). This phylogenomic-based scenario is compatible with a very early episode of reductive evolution on the economy side of the saddle manifold of the persistence triangle that streamlined the protist-like ancestors into primordial archaeal organisms. It appears these lineages were slow evolving and their diversity materialized late in evolution (Wang et al., 2007; Wang and Caetano-Anollés, 2009; Kim and Caetano-Anollés, 2011, 2012). It is also likely that the reductive episode was probably triggered by the harsh conditions of primordial Earth. A second episode of this kind resulted in the ancestors of Bacteria and the more efficient coverage of many new mesophilic niches. In contrast, the predator-induced push toward the flexibility and robustness side of the protistan saddle triggered the rise of Protista and then of the other eukaryal kingdoms, with diversity being attained quite late in evolution. Thus, phylogenomic trees describing the evolution of folds, FSFs and domain families and corresponding phylogenies describing the evolution of proteomes put forth scenarios of origin and evolution of the six kingdoms and three superkingdoms that are compatible with the broad generalization of the economy/flexibility/robustness persistence triangle.

## Conflict of Interest Statement

The authors declare that the research was conducted in the absence of any commercial or financial relationships that could be construed as a potential conflict of interest.

## Supplementary Material

The Supplementary Material for this article can be found online at http://www.frontiersin.org/Systems_Biology/10.3389/fgene.2013.00016/abstract

Supplementary Datasheet S1 **Motility speeds for single-celled organisms, metazoan, plants, and fungi**. Three tables of motility kinds, cellular actuators, medium appropriate for each motility kind, examples of organism species using each type of motility, the ranges of speed, and references.

Supplementary Datasheet S2 **Archaea tend to have sparser scope than bacteria**. Text of our argumentation as to why Archaea tend to have sparser scope than bacteria, with references.

Supplementary Datasheet S3 **Worksheet 1: a spreadsheet with the names of fully sequenced organisms, for which we obtained the data on FSF content, cell volume, and lifestyle**. Abbreviations: A, Archaea; B, Bacteria; E, Eukarya; F, Fungi; meta, Metazoa; proto, Protista; pla, Plantae. Each organism has a citation to the source of data on lifestyle and the source of data on cell volume, in separate columns. When a single source provided information on both, then only a single reference column contains the citation. Multiple sources of cell volume were given when data for mean, minimum and maximum volume could not be found in a single publication. All Metazoa are commonly known free-living species, and so lifestyle citations are not provided. NCBI refers to the NCBI genome project pages; JGI refers to the JGI finished genome pages. Worksheet 2: a spreadsheet with the names of fully sequenced Akarya, for which we obtained the data on nutritional preferences, motility, sporulation, pressure, pH, temperature, salinity and growth rate. Abbreviations: A, Archaea; B, bacteria. The data were obtained from the GOLD genomes online database, NCBI genome projects, and JGI genome projects. When data could not be obtained from those databases, books and published articles were used, as well as other databases. Citations are provided.

Supplementary Datasheet S4 **Methods**.
