# Supplementary material for: A General Framework of Persistence Strategies for Biological Systems Helps Explain Domains of Life
Source: Front Genet. 2013 Feb 25;4:16. doi: 10.3389/fgene.2013.00016 (PMC3580334; doi:10.3389/fgene.2013.00016)
Supplement: Supplementary Datasheet S1 — Motility speeds for single-celled organisms, metazoan, plants, and fungi. Three tables of motility kinds, cellular actuators, medium appropriate for each motility kind, examples of organism species using each type of motility, the ranges of speed, and references. [file 36588_Caetano-Anolles_DataSheet1.PDF]

Additional file 1

Table S1 | Motility speeds, cell sizes and life spans characteristic of single-celled organisms. The table describes single cell motility, presented in order of increasing speed, with examples from the kingdoms of Archaea, Bacteria and Protista.

Kinds of motility	Cellular actuators	Medium	Archaea examples	Bacteria examples	Protista examples	Speed	References
Buoyancy	vesicles filled with gas or liquids of low specific gravity	liquid	*Haloabacterium salinarium*	*Cyanobacteria*	diatoms		1, 14
Corkscrew/cell bending/peristalsis	periplasmic flagella (B) or fibrillar elements (E)	viscous matter	-	*Treponema primitia, Borrelia burgdorferi*	*Gregarines*	0.23-0.79 μ/sec	1, 2, 10
Gyration	periplasmic flagella,<br><br>external flagella	liquid		*Eberthella typhosa*	flagellates	Gyration results from flagellar motility	2, 8, 9
Twitching	pili	solid	-	*Neisseria gonorrhoeae*	-	0.05-1 μ/sec	1
Gliding	cell-surface adhesins (B) or membrane fold undulations (E)	solid	-	*Flavobacterium spp.*	*Gregarines*	0.03-11.1 μ/sec	1, 2, 3, 7
Inchworming or centipede-like	cell-surface Gli proteins	solid	-	*Mycoplasmas*	-	2-5 μ/sec	1, 13
Amoeboid	cytoskeletal filaments	solid	-	-	*Amoeba proteus*	5 μ/sec	4
Flagella-less swimming	unknown, possibly cell surface spicules	liquid	-	*Synechococcus*	-	5-25 μ/sec	1
Propagating kinks or helical waves	cytoskeletal filaments	viscous matter	-	*Spiroplasma spp.*	*Spirochaetes*	1-40 μ/sec	1, 2, 11, 12
Swim-and-tumble	external flagella	liquid	*Methanococcus maripaludis*	*Escherichia coli; Thiovulum majus* is the fastest known	-	2-615 μ/sec	1, 15
9+2 flagellar, with a large variety of subtypes	flagella	liquid	-	-	*Ceratium*	20-200 μ/sec	2, 4, 5
Cilial, with a large variety of subtypes	cilia	liquid	-	-	*Paramecium multimicronucleatum*	400-2000 μ/sec, and up to tens of mm/sec	2, 4, 6, 19

Dashes indicate that we have not been able to find evidence for a particular kind of motility in that kingdom. Examples are not exclusive: for example, cyanobacteria display not only buoyancy, but also gliding and swimming, and other bacteria are known to display buoyancy. B, Bacteria, E, Eukarya.

It would have been desirable to display the motility speed, cell size, and life span for each species of interest, and each kind of motility on Figure 2. However, all three parameters are not usually available simultaneously in the published literature. Thus, we tried to sample the literature for the data that would help us delineate scope size of each unicellular kingdom as a whole. The two tables below display the data used in Figure 2.

Data used in Figure 2A:

**Species**	**Cell size measurements, micrometers**	**Characteristic speed, micrometers per second**	**Reference**
Rhodobacter sphaeroides	2-2.5	5-40, with a mean of 11.2	[16]
Unclassified vibrioid bacteria	1.3-1.5 x 4-10	74.9+/-10.8	[17]
Escherichia coli	1.5x3	30	[18] Table 3, see references therein
Bdellovibrio bacteriovorus	0.25x1.4	140	[18] Table 3, see references therein
Thiospirillum jenese	4x40	20	[18] Table 3, see references therein
Spirochaeta litoralis	0.45x13	5.98	[18] Table 3, see references therein
Paramecium_multimicronucleatum	225	400-2000, with a mean of 1200	[6]
Amoeba proteus	500	5	[4]
Ceratium_tripos	225x332	250	[18] Table 4, see references therein
Euglena_gracilis	45x15	162	[18] Table 4, see references therein
Trypanosoma_cruzi	20x2	304	[18] Table 4, see references therein

Data used in Figure 2B:

**Species**	**Lifespan**	**Reference**
Colpidium striatum	13.56-40 hours	[20]
Ammonia beccarii	2-3 months	[21] Table 1, see references therein
Amphistegina lessonii	4-12 months	[21] Table 1, see references therein
Elphidium crispun	1 year	[21] Table 1, see references therein
Spirillina vivipara	10 days	[21] Table 1, see references therein
Nummulites laevigatus	>=5 years	[22]
Dictyostellium discoideum amoebae	3-24 hours	[23]
Physarium polycephalum amoebae	2-4 days	[24]
Paramecium aurelia	4-24 hours	[25]

This information is based on the following works:

1. Jarrell KF, McBride MJ: The surprisingly diverse ways that prokaryotes move. Nature reviews 2008, 6:466-476
2. Jahn TL, Votta JJ: Locomotion of Protozoa. Annu. Rev. Fluid Mech. 1972, 4:93-116
3. Ward MJ, Zusman DR: Regulation of directed motility in Myxococcus xanthus. Molecular Microbiology 1997, 24(5):885-893
4. Alexander RM: *The invertebrates*. Cambridge University Press; London, UK 1979
5. Mitchell DR: The evolution of eukaryotic cilia and flagella as motile and sensory organelles. In *Origins and Evolution of Eukaryotic Endomembranes and Cytoskeleton*. Landes Bioscience; 2006:130-140
6. Dennis B: *Cell movements*. Garland, New York, NY; 1992
7. Haupt W, Feinleib ME: *Encyclopedia of plant physiology*. New Series v.7. Springer-Verlag, Berlin; 1979
8. Pijper A: Shape and motility of bacteria. J. Path. Bact. 1946, 58: 325-342
9. Pijper A: Methylcellulose and bacterial motility. J. Bacteriol. 1947, 53(3):257-269
10. Pietrantonio F, Noble P.B, Amsel R, Chan ESC: Locomotory characteristics of *Treponema denticola*. Can. J. Microbiol. 1988, 34:748-752
11. Li C, Wolgemuth CW, Marko M, Morgan DG, Charon NW: Genetic analysis of spirochete flagellin proteins and their involvement in motility, filament assembly, and flagellar morphology. J Bacteriol. 2008 Aug 190(16):5607-5615
12. Daniels MJ, Longland JM, Gilbart J: Aspects of motility and chemotaxis in Spiroplasmas. Journal of general microbiology 1980, 118:429-436
13. Miyata M: Centipede and inchworm models to explain *Mycoplasma* gliding. Cell trends in microbiology 2007, 16(1):6-12
14. Barsanti L, Gualtieri P: Algae: anatomy, biochemistry and biotechnology. CRC Press, Boca Raton, FL; 2006
15. Garcia-Pichel F: Rapid bacterial swimming measured in swarming cells of *Thiovulum majus.* Journal of Bacteriology 1989, 171(6):3560-3563
16. Packer HL, Armitage JP: The Chemokinetic and Chemotactic Behavior of *Rhodobacter sphaeroides*: Two Independent Responses. Journal of Bacteriology 1994,176(1):206-212
17. Thar R, Kühl M: Conspicuous Veils Formed by Vibrioid Bacteria on Sulfidic Marine Sediment. Applied and Environmental Microbiology 2002, 68(12):6310-6320
18. Brennen C, Winet H: Fluid mechanics of propulsion by cilia and flagella. Ann. Rev. Fluid Mech. 1977, 9:339-98
19. Vandromme P, Schmitt FG, Souissi S, Buskey EJ, Stickler JR, Wu C-H, Hwang J-S: Symbolic analysis of plankton swimming trajectories: case study of *Strobilidium* sp. (Protista) helical walking under various food conditions. Zoological Studies 192010, 49(3):289-303
20. Elliott AM: Isolation of *Colpidium striatum* Stokes in bacteria-free cultures and the relation of growth to pH of the medium. Biological Bulletin 1933, 65(1):45-56
21. Hallock P: Why are larger foraminifera large? Paleobiology 1985, 11(2):195-208
22. Purton LMA, Braiser MD: Giant protist *Nummulites* and its Eocene environment: life span and habitat insight from δ^{18}O and δ^{13}C data from *Nummulites* and *Venericardia*, Hampshire basin, UK. Geology 1999, 27(8):711-714
23. Devreotes P: *Dictyostellium discoideum*: a model system for cell-cell interactions on development. Science 1989, 245(4922):1054-1058
24. McCullough CHR, Dee J: Defined and semi-defined media for the growth of amoebae of *Physarum polycephalum*. Journal of General Microbiology 1976, 95:151-158
25. Smith-Sonneborn J, Reed JC: Calendar life-span versus fission life-span of *Paramecium aurelia.* Journal of Gerontology 1976, 31(1):2-7

Table S2 | Motility speeds, linear body sizes and life spans characteristic of metazoan. The table below describes motility observed in Metazoa, presented in order of increasing speed, with relevant examples, some of which were also used in Figure 3A. In most cases motility is powered by musculature, and therefore is faster for bigger organisms due to more powerful actuators and bigger leverage provided by longer limbs. The focus of this table is on the kinds of motility that spatially displace the entire body of the organism, not those that bring food to the mouth, or to stir the surrounding medium. All speeds are converted to microns per second, for comparison with Tables S1 and S3.

Kinds of motility	Mechanism	Medium	Metazoa examples	Linear size	Speed	References
Ciliary walking	ciliated ventral cells	solid	*Trichoplax*	120-500 μm	1-10 μm/sec, with peaks up to 25 μm/sec	1, 2
Worm-like	longitudinal alternations of swelling and contractions of circumferential musculature	solid	Nematodes	~1 mm	average 31-232 μm/sec, with peaks up to 400 μm/sec	3
Gliding on a large foot	waves of muscular contraction of the foot surface and/or cilia in combination with mucus	solid	*Helix lucorum*<br><br>*Lymnaea peregra*	5-42 mm<br><br>5-20 mm	200-600 μm/sec<br><br>average 16.6-350 μm/sec depending on the substrate, with maximum speed at 633 μm/sec	4, 5<br><br>6, 7, 8
Inchworming, vermiforming	alternating suction on the extremities	solid	Medicinal leech	6-10 cm	On average $1.28\text{-}1.30 \cdot 10^4$ μm/sec	9
Caterpillar-like	waves of longitudinal muscle contraction, coupled with muscle-actuated appendage attachment and detachment	solid	Caterpillars	centimeters	up to $2.85 \cdot 10^4$ μm/sec	10, 11
Active rolling	anterior-posterior cartwheeling by pushing off the substrate with the most peripheral appendages	solid	Mother of Pearl caterpillar, *Nannosquilla decemspinosa*, Pangolins	-	$(3.5\text{-}39) \cdot 10^4$ μm/sec	12, 13
Lateral body undulation	posteriorly moving waves push against the contact points on the substrate	Solid or liquid	Garter snakes<br>Herring<br>Salmon<br>Swordfish<br>Sailfish	14.9-66 cm<br>0.3 m<br>1 m<br>4 m<br>3.4 m	cruise $(33\text{-}46) \cdot 10^3$ μm/sec, burst $(27\text{-}110) \cdot 10^4$ μm/sec<br>$1.67 \cdot 10^6$ μm/sec<br>$12.5 \cdot 10^6$ μm/sec<br>$26.7 \cdot 10^6$ μm/sec<br>bursts up to $3.1 \cdot 10^7$ μm/sec	14, 15<br>16<br>16, 17<br>16<br>18

Kinds of motility	Mechanism	Medium	Metazoa examples	Linear size	Speed	References
			Blue whale<br>Wahoo	26 m<br>1.1 m	$10.3 \cdot 10^6$ μm/sec<br>$21.5 \cdot 10^6$ μm/sec	19<br>19
Passive rolling	gravity acting on the animal curled into a ball or using its appendages like a cartwheel	solid	Web-toed salamanders, Wheeling spiders, Pangolins	-	$(1–3) \cdot 10^6$ μm/sec	20, 21, 22
Jet propulsion	squirting jets of water out of soft mantle through a siphon	liquid	Cephalopods; squid is the fastest	Centimeters to meters	$0.30 \cdot 10^6$ μm/sec during sustained swimming and up to $9 \cdot 10^6$ μm/sec maximum	23, 24
Limbed locomotion	muscle-actuated movement of appendages	solid	Cheetah<br>Ant<br>Clover mite	2.13 m<br>4.2 mm<br>0.8 mm	Sustained at (1.2-1.7) 10^7 μm/sec, bursts at $2.5 \cdot 10^7$ μm/sec<br>$6.5 \cdot 10^4$ μm/sec<br>$8.5 \cdot 10^3$ μm/sec	25<br>19<br>19
		air	Bumble bee<br>Storm petrels<br>The Great Auks<br>Swifts<br>Arctic tern<br>Dragonfly<br>Fruit fly	~1.5 cm<br>13-26 cm<br>15 cm<br>9-25 cm<br><br>8.5 cm<br>2 mm	$4.9 \cdot 10^6$ μm/sec<br>ground speeds $5.5 \cdot 10^6$ μm/sec<br>$22.3 \cdot 10^6$ μm/sec<br>speeds from $4.4 \cdot 10^6$ to $26.9 \cdot 10^6$ μm/sec in normal flight<br>cruise $5\text{-}14 \cdot 10^6$ μm/sec, bursts up to $47.5 \cdot 10^6 \cdot$μm/sec<br>$10 \cdot 10^6 \cdot$μm/sec<br>$1.910^6 \cdot$μm/sec	26<br>26, 27<br>26, 27<br>28<br>29<br>19<br>19
Tooled locomotion	Fuel-powered machine engines	any	Man	~1.7 m	Walking speed $1.19\text{-}1.51 \cdot 10^6$ μm/sec<br>Apollo 8 shuttle during take-off in air attained maximum speed of $1.1 \cdot 10^{10}$ μm/sec	30, 31

Data referred used in Figure 2B:

Organism	Spatial Range	Life span	References
Cheetah	~11-26 km	5-9 years	25, 32, 33, 34,
Canis lupus	200 km dispersal	~ 10 years	35
Elephants	3-93 km	<70 years	36, 37
Swifts	~1800 km	5.1-25 yrs	38, 39
Arctic tern	~30000km	<=34years	28, 40
Sparrows	~180-560 km	3-13 yrs	29, 41
Tanagers	850-2360 km	5-9.5 yrs	42
Bobolinks	1490-2340 km	6-11 yrs	43
Warblers	540-2160 km	3-11 years	42
Vireos	560-2070 km	13 years	44
Crocodylus porosus	<=126 km	41.7 years	45
Humpback whale	8300 km	>48 years	46, 47
Martes Americana	2.6 km	<=15 years	48, 49
Martes pennant	3.7 km	~10 years	50, 51
Lynx rufus	up to ~6.5 km	~4.5 years	50, 52
Gulo gulo	average of 20.5, up to 100	8-10 years	50, 53
Bowhead whales	km	<=211 years	50, 54, 55
Sauromalus obesus	158 km	9.3 years	50, 56, 57
Cnemidophorus tigris	110-295 m	7.8 years	58, 59
Lygosoma laterale	78.9-92.8 m	3.6-4 years	58, 59
Dipsosaurus dorsalis	9-15.9 m	14.6 years	57, 59
Conolophys pallidus	25.3m – 25.9 m	17.1 years	59, 61
Varanus gouldii	53.7-92.8 m	18.3 years	59, 61
Man	280.2m	67.2 years	59, 61
	400,171 km		30, 31, 62, 63

The information in these tables is based on the following works:

1. Ueda T, Koya S, Maruyama YK (1999) **Dynamic patterns in the locomotion and feeding behaviors by the placozoan *Trichoplax adhaerence*.** Biosystems **54**:65-70
2. Pearse VB, Voigt O: **Field biology of placozoans (*Trichoplax*): distribution, diversity, biotic interactions.** Integrative and comparative biology 2007, **47**(5):677-692
3. Ramot D, Johnson BE, Berry TL Jr, Carnell L, Goodman MB (2008) **The Parallel Worm Tracker: A Platform for Measuring Average Speed and Drug-Induced Paralysis in Nematodes.** PLoS ONE 3(5): e2208

4. Pavlova GA (2001) **Effects of serotonin, dopamine and ergometrine on locomotion in the pulmonate mollusc *Helix lucorum*.** J. Exp. Biol. **204**:1625-1633
5. Staikou A, Lazaridou-Dimitriadou M, Farmakis N: **Aspects of the life cycle, population dynamics, growth and secondary production of the edible snail *Helix lucorum Linnaeus*, 1758 (Gastropoda, pulmonata) in Greece.** J. Moll. Stud. 1988, **54:**139-155
6. Storey R (1971) **Some observations on the feeding habits of Lymnaea peregra (Müller)**. Proc. Malac. Soc. Lond. **39**, 327
7. Lam PKS, Calow P: **Intraspecific life-history variation in *Lymnaea peregra* (Gastropoda:Pulmonata). I. Field study.** Journal of animal ecology 1989, **58**(2):571-588
8. Lam PKS, Calow P: **Differences in the shell shape of *Lymnaea peregra* (Müller) (gastropoda: pulmonata) from lotic and lentic habitats; environmental or genetic variance?** J. Moll. Stud. 1988, **54:**197-207
9. Stern-Tomlinson W, Nusbaum MP, Perez LE, Kristan WB Jr. (1986) **A kinematic study of crawling behavior in the leech, *Hirudo medicinalis*.** J. Comp. Physiol. A **158**:593-603
10. Chapman RF (1998) The insects: structure and function. Cambridge University Press, Cambridge, UK
11. Stulce JR (2002) Conceptual Design and Simulation of a Multibody Passive-Legged Crawling Vehicle. Retrieved February 13 2011 from Electronic Thesis Database; etd-04262002-012250
12. Brackenbury J (1999) **Fast locomotion in caterpillars.** J. Insect Physiol. **45**:525-533
13. Caldwell RL (1979). **A unique form of locomotion in a stomatopod — backward somersaulting.** Nature **282**: 71–73
14. Heckrotte C (1967) **Relations of body temperature, size, and crawling speed of the common garter snake, *Thamnophis s. sirtalis*.** Copeia **4**:759-763
15. Jayne BC, Bennett AF: **Scaling of speed and endurance in garter snakes: a comparison of cross-sectional and longitudinal allometries.** J. Zool., Lond. 1990, **220:**257-277
16. National Maritime Research Institute, Fish swimming speeds database [http://www.nmri.go.jp/eng/khirata/fish/general/speed/speede.htm]
17. Robertson OH: **Prolongation of the life span of kokanee salmon (Oncorhyncus nerka kennerlyi) by castration before beginning of gonad development.** Zoology 1961, **47:**609-621
18. Wolter C, Arlinghaus R: **Burst and critical smiwwimg speeds of fish and their ecological relevance in waterways.** Leibniz-Institute of Freshwater Ecology and Inland Fisheries Annual Report 2003:77-93
19. McMahon TA, Bonner JT: *On size and life.* Scientific American Books, Inc. New York. 1983
20. Garcia-Paris M, Deban SM (1995) **A novel antipredator mechanism in salamanders: rolling escape in *Hydromantes platycephalus*.** J ournal of Herpetology **29**:149-151
21. Henschel, J.R. (1990). **Spiders wheel to escape**. South African Journal of Science **86**: 151–152
22. Tenaza RR (1975) **Pangolins rolling away from predatory risks.** Journal of Mammalogy **56**:257
23. Anderson EJ, DeMont ME: **The mechanics of locomotion in the squid *Logilo pealei*: locomotory function and unsteady hydrodynamics of the jet and intramantle pressure.** J. Exp. Biol. 2000 **203**:2851-2863
24. Gosline JM, DeMont ME (1985) **Jet-propelled swimming in squids.** Sci. Amer. **256**:96-103
25. Hildebrand M (1961) **Further studies on locomotion of the cheetah.** Journal of mammalogy. **42**(1):84-91
26. Meinertzhagen R: **The speed and altitude of bird flight (with notes on other animals).** IBIS 1955, **97**(1):81-117
27. Spear LB, Ainley DG: **Flight speed of seabirds in relation to wind speed and direction.** IBIS 1997, **139**(2):234-251
28. Odum EP, Connell CE, Stoddard HL: **Flight energy and estimated flight ranges of some migratory birds.** The Auk 1961, **78**(4):515-527
29. Egevang C, Stenhouse IJ, Phillips RA, Petersen A, Fox JW, Silk JRD: **Tracking of Arctic terns *Sterna paradisaea* reveals longest animal migration.** PNAS 2010, **107**(5):2078-2081
30. NASA: From "Apollo 8 Man Around the Moon," NASA EP-66, Office of Public Affairs, NASA Headquarters, Washington, D.C. 20546. [http://er.jsc.nasa.gov/seh/apollo8.html; 2010]
31. Knoublauch RL, Pietrucha MT, Nitzburg M: **Field studies of pedestrian walking speed and start-up time.** Transportation Research Record 1996, **1538**:27-38

32. Kelly MJ, Laurenson MK, FitzGibbon CD, Collins DA, Durant SM, Frame GW, Bertram BCR, Caro TM: **Demography of the seremgeti cheetah (*Acinonyx jubatus*) population: the first 25 years.** J. Zool., Lond. 1998, **244:**473-488
33. Laurenson MK: **Implications of high offspring mortality for cheetah population dynamics.** In: Serengeti II: dynamics, management, and conservation of an ecosystem. Chicago Press, Chicago; 1995
34. Broomshall LS, Mills MGL, du Toit JT: **Home range and habitat use by cheetahs (*Acinonyx jubatus*) in the Kruger National Parl.** J. Zool., Lond. 2003, **261**:119-128
35. Houser A, Somers MJ, Boast LK: **Home range use of free-ranging cheetah on farm and conservation land in Botswana.** South African Journal of Wildlife Research 2009, **39**(1):11-22
36. Hefner R, Geffen E: **Group size and home range of the Arabian wolf (*Canis lupus*) in Southern Israel.** Journal of Mammalogy 1999, **80**(2)611-619
37. Lovari S, Sforzi A, Scala C, Fico R: **Mortality parameters of the wolf in Italy: does the wolf keep himself from the door?** Journal of Zoology 2007, **272**:117-124
38. Osborn FV: **The concept of home range in relation to elephants in Africa.** Pachyderm 2004, **37**:37-44
39. Age criteria for the african elephant loxodonta a. Africana R. M. Laws; African journal of ecology volume 4, issue 1, pages 1–37, 1966
40. Botkin DB, Miller RS: **Mortality rates and survival of birds.** The American Naturalist 1974, **108**(960):181-192
41. Hatch JJ: **Longevity record for the Arctic Tern**. Bird-Banding 1974, **45**:269-270
42. Klimkiewicz MK, Futcher AG: **Longevity records of North American birds: Coerebinae through Estrildidae.** Journal of Field Ornithology 1987, **58**(3):318-333
43. Snow DW, Lill A: **Longevity records for some neotropical land birds.** The Condor 1974, **76**(3):262-267
44. Klimkiewicz MK, Clapp RB, Futcher AG: **Longevity records of North American Birds: Remizidae through Parulinae.** Journal of Field Ornithology 1983, **54**(3):287-294
45. Woodsworth BL: **Survival and longevity of the Puerto Rican Vireo.** The Wilson Bulletin 1999, **111**(3):376-380
46. Bowler, J.K., 1975. Longevity of reptiles and amphibians in N. American collections as of 1 November, 1975. Society for the Study of Amphibians and Reptiles, Miscellaneous Publications, Herpetological Circular 6:1-32.
47. Read MA, Grigg GC, Irwin SR, Shanahan D, Franklin CE: **Satellite tracking reveals long distance coastal travel and homing by translocated estuarine crocodiles, *Crocodylus porosus.*** PLOS One 2007, **9**:e929
48. Rasmussen K, Palacios DM, Calambokidis J, Saborio MT, Dall Rosa L, Secchi ER, Steiger GH, Allen JM, Stone GS: **Southern hemisphere humpback whales wintering off Central America: insights from water temperature into the longest mammalian migration.** Biol. Lett. 2007, **3**:302-305
49. Mann J, Connor RC, Tyack PL, Whitehead H: **Cetacean societies. Field studies of dolphins and whales.** The University of Chicago Press, Chicago; 2000
50. Lindstedt SL, Miller BJ, Buskirk SW: **Home range, time, and body size in mammals.** Ecology 1986, **67**(2):413-418
51. Buskirk SW, Ruggiero LF: **American marten.** In: The Scientific Basis for Conserving Forest Carnivores. American marten, fisher, lynx, and wolverine. USDA Forest Service General Technical Report RM-254; 1994
52. Powell RA, Zielinski WJ: **Fisher.** In: The Scientific Basis for Conserving Forest Carnivores. American marten, fisher, lynx, and wolverine. USDA Forest Service General Technical Report RM-254; 1994
53. Koehler GM, Aubry KB: **Lynx.** In: The Scientific Basis for Conserving Forest Carnivores. American marten, fisher, lynx, and wolverine. USDA Forest Service General Technical Report RM-254; 1994
54. Banci V: **Wolverine.** In: The Scientific Basis for Conserving Forest Carnivores. American marten, fisher, lynx, and wolverine. USDA Forest Service General Technical Report RM-254; 1994
55. Groves CR: **Distribution of the wolverine in Idaho as determined by mail questionnaire.** Northwest Science 1988, **62**(4):181-185
56. George JC, Bada J, Zeh J, Scott L, Brown SE, O'Hara TO, Suydam R: **Age and growth estimates of bowhead whales (*Balaena mysticetus*) via aspartic acid racemization.** Can. J. Zool. 1999, **77**:571-580
57. Heide-Jorgensen MP, Laidre K, Borchers D, Samarra F: **Surprising recovery of bowhead whales.** In *Proceedings of the International Whaling Commission annual meeting 2007.* [http://www.iwcoffice.org/_documents/sci_com/SC59docs/SC-59-BRG23.pdf]

58. Turner FB, Jennrich RI, Weintraub JD: **Home ranges and body size of lizards.** Ecology 1969, **50**(6):1076-1081
59. Human aging resources database [http://genomics.senescence.info/]
60. Brooks GRJr: **Population ecology of the Ground skink, Lygosoma laterale (Say).** Ecological monographs 1967, **37**(2):71-87
61. Christian KA, Waldschmidt S: **The relationship between lizard home range and body size: a reanalysis of the data.** Herpetologica 1984, **40**(1):68-75
62. NASA space missions [http://www.nasa.gov/mission_pages/apollo/missions/apollo13.html]
63. NASA the world factbook [https://www.cia.gov/library/publications/the-world-factbook/rankorder/2102rank.html]

Table S3 | Motility speeds for plantae and fungi. The table below describes ways of movement in plantae and fungi, presented in order of increasing speed, with examples.

Kinds of motility	Actuators	Organism examples	Linear Size	Speed	References
Growth in substrate	Vacuolar pressure within apical cells on the growing tips of fungal colonies and plants	*Aspergillus nidulans* hyphal tip	233 µm unbranched hyphal length	27.48 µm/sec	1,2
		Neurospora crassa leading hyphae	59.26 µm hyphal length	2280 µm/sec	3-5
		Coprinus disseminatus apical hyphae	341 µm interseptal length	0.0067-0.078 µm/sec	8,9
		Fusarium culmorum		0.06-0.13 µm/sec	10
		Gilbertella persicaria		0.25-0.37 µm/sec	10
		Pythium aphanidermatum		0.21-0.30 µm/sec	10
		Rhizoctonia solani		0.04-0.11 µm/sec	10
		Trichoderma viride		0.16-0.29 µm/sec	10
		Saprolegnia ferax		0.13-0.29 µm/sec	10
		Lettuce seedling root tips	~ 3 cm total seedling length	0.13-0.14 µm/sec	6
		Maize seedling root tips	~3.5-5 cm seedling root length	up to 0.5 µm/sec	7

The information in this table is based on the following works:

1. Horio T, Oakley BR: **The role of microtubules in rapid hyphal tip growth of *Aspergillus nidulans*.** Molecular Biology of the Cell 2005, **16**:918-926
2. Dynesen J, Nielsen J: **Branching is coordinated with mitosis in growing hyphae of Aspergillus nidulans.** Fungal Genetics and Biology 2003, **40**:15-24
3. Fleißner A, Sarkar S, Jacobson DJ, Roca MG, Read ND, Glass NL: **The so Locus Is Required for Vegetative Cell Fusion and Postfertilization Events in Neurospora crassa.** Eukaryotic Cell 2005, **4**(5):920-930
4. Martegani E, Tome F, Trezzp F: **Timing of nuclear division cycle in *Neurospora crassa*.** J. Cell Sci. 1981, **48**:127-136
5. Steele GC, Trinci APJ: **The extension zone of mycelial hyphae.** New Phytologist 1975, **75**(3):583-587
6. Tanimoto E, Watanabe J: **Automated recording of lettuce root elongation as affected by auxin and acid pH in a new rhizometer with minimum mechanical contact to roots.** Plant Cell Physiol. 1986, **27**(8):1475-1487
7. Shimazaki Y, Ookawa T, Hirasawa T: **The root tip and accelerating region suppress elongation of the decelerating region without any effects on cell turgor in primary roots of maize under water stress.** Plant physiology 2005, **139**:458-465
8. Butler GM: **Growth of hyphal branching systems in *Coprinus disseminatus.*** Annals of Botany 1961, **25**(99):341-352
9. Butler GM: **Effects of autoinhibition on hyphal growth in *Coprinus disseminatus.*** Trans. Br. Mycol. Soc. 1984, **83**(1):131-137
10. Lopez-Franco R, Bartnicki-Garcia S, Bracker CE: **Pulsed growth of fungal hyphal tips.** Proc. Natl. Acad. Sci. USA 1994, **91**:12228-12232
