# Supplementary material for: A General Framework of Persistence Strategies for Biological Systems Helps Explain Domains of Life
Source: Front Genet. 2013 Feb 25;4:16. doi: 10.3389/fgene.2013.00016 (PMC3580334; doi:10.3389/fgene.2013.00016)
Supplement: Supplementary Datasheet S2 — Archaea tend to have sparser scope than bacteria. Text of our argumentation as to why Archaea tend to have sparser scope than bacteria, with references. [file 36588_Caetano-Anolles_DataSheet2.PDF]

Additional file 2

ARCHAEA TEND TO HAVE SPARSER SCOPE THAN BACTERIA

Archaea tend to avoid highly variable environments. For example, Archaea cohabit with Bacteria within the highly patterned geothermal sites, but they tend to be found in greater abundances within the more thermally and chemically stable chimneys (Harmsen et al., 1997; Takai and Horikoshi, 1999; Takai et al., 2001). The hydrothermal plume is dominated by bacteria, which probably experience a wide range of thermal conditions, being carried by the turbulently mixing flow, falling down to the cool sediment, stirred up and possibly carried by indigenous metazoa back into the hot plume (Naganuma et al., 1989; Takai et al., 2004). Within the plume, Archaea are present in very low numbers, but their abundance is increased in the ambient water outside the plume (Takai et al., 2004) – likely a somewhat calmer, less turbulent area. Akarya are also present in noticeable quantities deep within the stable oceanic and freshwater sediments (Nealson, 1997; Takami, 1997; Marchesi, 2001; Cowen et al., 2003; Newberry et al., 2004; Liao et al., 2009; Koizumi et al., 2003; Purdy et al., 2003; Chernitsina et al., 2007; Schwarz et al., 2007; Teske and Sorensen, 2008; Tao et al., 2008; Xiangchum et al., 2009). In contrast, it is bacteria that inhabit the sediment-ocean interface, receiving nutrition in form of organic material precipitating out of the water column – a variable nutrient source, providing a feast-and-famine lifestyle (Takami et al., 1997; Dixon and Turley, 2001) and a denser scope than deeper in the sediment. Our third example is the pelagic zone, where akarya can be exposed to significant gradients when carried by currents, or attached to animal bodies, leaves, etc. Archaeal presence wanes toward the surface (de Corte et al., 2009), and that of bacteria rises (Glöckner et al., 1999; Jurgens et al., 2000; Karner et al., 2001; DeLong, 2003; Martin-Cuadrado et al., 2007). However, the epipelagic zone is different. Active surface mixing regularly transports microorganisms between the warm, illuminated surface waters and the deeper, cooler, darker subsurface layers. Such variability encourages mixotrophic metabolism, which permits bacteria (Eiler, 2006) to switch circumstantially between photosynthesis and heterotrophy and form complex food webs (Moran and Miller, 2007; Sherr and Sherr, 2009), all evidence of well-developed flexibility mechanisms. Only low quantities of Archaea have been reported (DeLong, 2003) to inhabit surface waters (Tittel et al., 2003; Protasov, 2008). Another example of the highly variable environment avoided by Archaea is the epilithic niche on the surfaces of natural and construction rocks. This is a ubiquitous environment (Hoppert et al., 2004), but generally not a hospitable one (Walker and Pace, 2007). Rock surfaces do not provide the nutritive substrate and protection that the particles of soil do. Epilithic niches, particularly in the cold and hot deserts, are desiccated by wind (Knowles and Castenholz, 2008), are subject to great thermal disturbances (Waragai, 1998; Omelon et al., 2003; McKay et al., 1985, 1993, 2009, Garvie et al., 2008) and harbor high concentrations of salt and acid in the cracks and crevices inhabited by microorganisms (De los Rios et al., 2003; Walker et al., 2005; Wierzchos et al., 2006). Periodic exfoliation is another source of disturbance in this niche (Sun and Friedmann, 1999). At the same time, these epilithic environments perhaps create one of the very few niches conducive to life in the severe climates of hot and cold deserts, such as Atacama and Antarctica, by harboring residual water in the rock surface crevices. They are populated by lichens and symbiotrophic communities of fungi and cyanobacteria. Those inhabiting the frigid deserts of Antarctica have been shown to be the slowest-growing communities on Earth (Johnston and Vestal, 1991; Sun and Friedman, 1999) and are therefore exposed to the extremely variable environmental and nutritional signals

throughout their lifetimes. Endolithic Archaea have only been found in rocks positioned within mesophilic environments, where these variations are less extreme (Walker et al., 2005). Finally, Bacteria, but no Archaea, have been found in the highly disturbed and extremophilic environment of the atmosphere, with its turbulent currents, radiation, desiccation and temperature variability (Christner et al., 2008a; Christner et al., 2008b; Yang et al., 2009).

The greater density of the bacterial scope environment, compared to Archaea, is also reflected at the nutritional level. In Table 4 we name examples of archaeal and bacterial species for each strictotrophic and a number of mixotrophic nutritional categories. This table is not exhaustive when it comes to mixotrophs, and is only meant to illustrate the differences between the metabolic strategies of the two kingdoms. Immediately apparent is the metabolic diversity of bacteria, which seem capable of implementing virtually any nutritional category and are avid mixotrophs (Oren, 2006). Versatility of bacterial species is found not only in the number of nutritional categories, but also in the number of substrates an organism can use. Purple bacteria stand as a stark example. *Rhodopseudomonas* sp. can use lactate, malate, butyrate or acetate as sources of carbon (Barbosa et al., 2001). *Rhodopseudomonas palustris* uses thiosulfate, hydrogen gas, sulfur compounds, and possibly CO and formate as electron donors in respiration (Larimer et al., 2004). A purple sulfur bacterium *Allochromatium vinosum* is able to use hydrogen, sulfide, thiosulfate, sulfur and sulfite as electron donors, and formate, propionate, furamate, succinate, malate and glyconate as sources of carbon (Kumar et al., 2008). This versatility suggests that bacteria tend to occupy niches where nutrients change frequently, increasing bacterial scope density and content. We have not been able to find examples of archaeal species for many mixotrophic categories.

REFERENCES

Barbosa, M.J., Rocha, J.M.S., Tramper, J., and Wijffels, R. H. (2001). Acetate as a carbon source for hydrogen production by photosynthetic bacteria. *J. Biotechnol.* 85, 25-33.

Chernitsina, S.M., Zemskaya, T.I., Vorob'eva, S.S., Shubenkova, O.V., Khlystov, O.M., and Kostornova, T.Y. (2007). Comparative molecular biological analysis of the microbial community of the Holocene and Pleistocene deposits of Posol'skaya Shoal, Lake Baikal. *Microbiology* 76, 102-111.

Christner, B.C., Morris, C.E., Foreman, C. M., Cai, R., and Sands, D. C. (2008a). Ubiquity of biological ice nucleators in snowfall. *Science* 319, 1214.

Christner, B.C., Cai, R., Morris, C.E., McCarter, K.S., Foreman, C.M., Skidmore, M. L., Montross, S.N. and Sands, D.C. (2008b). Geographic, seasonal, and precipitation chemistry influence on the abundance and activity of biological ice nucleators in rain and snow. *Proc. Natl. Acad. Sci. USA* 105, 18854-18859.

de Corte, D., Yokokawa, T., Varela, M.M., Agogue, H., and Herndl, G. J. (2009). Spatial distribution of Bacteria and Archaea and amoA gene copy numbers throughout the water column of the Eastern Mediterranean sea. *ISME J.* 3, 147-158.

Cowen, J.P., Giovannoni, S.J., Kenig, F., Johnson, H.P., Butterfield, D., Rappe, M.S., Hutnak, M., and Lam, P. (2003). Fluids from aging ocean crust that support microbial life. *Science* 299, 120-123.

DeLong, E.F. (2003). Oceans of archaea. ASM News 69(10):503-511.

Dixon, J.L., and Turley, C.M. (2001). Measuring bacterial production in deep-sea sediments using ^{3}H-thymidine incorporation: ecological significance. *Microbial Ecol.* 42, 549-561.

Eiler, A. (2006). Evidence for the ubiquity of mixotrophic bacteria in the upper ocean: implications and consequences. *Appl. Environ. Microbiol.* 72, 7431-7437.

Garvie, L.A.J., Knauth, L.P., Bungartz, F., Knolowski, S., and Nash III T. H. (2008). Life in extreme environments: survival strategy of the endolithic desert lichen *Verrucaria rubrocincta*. *Naturwissenschaften* 95, 705-712.

Glöckner, F.O., Fuchs, B.M., and Amann, R. (1999). Bacterioplankton compositions of lakes and oceans: a first comparison based on fluorescence in situ hybridization. *Appl. Environ. Microbiol.* 65, 3721-3726.

Harmsen, J.M., Prieur, D., and Jeanthon, C. (1997). Distribution of microorganisms in deep-sea hydrothermal vent chimneys investigated by whole-cell hybridization and enrichment culture of thermophilic subpopulations. *Appl. Environ. Microbiol.* 63, 2876-2883.

Hoppert, M., Flies, C., Pohl, W., Gunzl, B., and Schneider, J. (2004). Colonization strategies of lithobiontic microorganisms on carbonate rocks. *Environ. Geol.* 46, 421-428.

Johnston, C.G., and Vestal, J.R. (1991). Photosynthetic carbon incorporation and turnover in Antarctic cryptoendolithic microbial communities: are they the slowest-growing communities on Earth? *Appl. Environ. Microbiol.* 57, 2308-2311.

Jurgens G., Glöckner, F.O., Amann, R., Saano, A., Montonen, L., Likolammmi, M., and Münster, U. (2000). Identification of novel Archaea in bacterioplankton of a boreal forest lake by phylogenetic analysis and fluorescent in situ hybridization. *FEMS Microbiol. Ecol.* 34, 45-56.

Karner, M.B., DeLong, E.F., and Karl, D.M. (2001). Archaeal dominance in the mesopelagic zone of the Pacific Ocean. *Nature* 409, 507-510.

Knowles, E.J., and Castenholz, R. W. (2008). Effect of exogenous extracellular polysaccharides on the desiccation and freezing tolerance of rock-inhabiting phototrophic microorganisms. *FEMS Microbiol. Ecol.* 66, 261-270.

Koizumi, Y., Takii, S., Nishino, M., and Nakajima, T. (2003). Vertical distributions of sulfate-reducing bacteria and methane-producing archaea quantified by oligonucleotide probe hybridization in the profundal sediment of a mesotrophic lake. *FEMS Microbiol. Ecol.* 44, 101-108.

Kumar, P.A., Srinivas, T.N.R., Sasikala, C., and Ramana, C.V. (2008). *Allochromatium renukae sp. nov*. *Intl. J. Syst. Evol. Microbiol.* 58, 404-407.

Larimer, F.W., Chain, P., Hauser, L., Lamerdin, J., Malfatti, S., Do, L., Land, M.L., Pelletier, D.A., Beatty, J.T., Lang, A.S., Tabita, F.R., Gibson, J.L., Hanson, T. E., Bobst, C., Torres, J.L,. Torres, Y., Peres, C., Harrison, F. H., Gibson, J., and Harwood, C.S. (2004). Complete genome sequence of the metabolically versatile photosynthetic bacterium *Rhodopseudomonas palustris*. *Nature Biotechnol.* 22, 55-61.

Liao, L., Xu, X., Wang, C., Zhang, D., and Wu, M. (2009). Bacterial and archaeal communities in the surface sediment from the northern slope of the South China Sea. *J. Zhejiang University Science B* 10, 890-901.

Marchesi, J.R., Weightman, A.J., Cragg, B.A., Parkes, R.J., and Fry, J.C. (2001). Methanogen and bacterial diversity and distribution in deep gas hydrate sediments from the Cascadia Margin as revealed by 16S rRNA molecular analysis. *FEMS Microbiol. Ecol.* 34, 221-228.

Martin-Cuadrado, A., Lopez-Garcia, P., Alba, J., Moreira, D., Monticelli, L., Strittmatter, A., Gottschalk, G. and Rodriguez-Valera, F. (2007). Metagenomics of the deep mediterranean, a warm bathypelagic habitat. *PLoS ONE* 9, e914.

McKay, C.P., and Friedmann, E.I. (1985). The cryptoendolithic microbial environment in the Antarctic cold desert: temperature variations in nature. *Polar Biol.* 4, 19-25.

McKay, C.P., Nienow, J.A., Meyer, M.A., and Friedmann, E.I. (1993). Continuous nanoclimate data (1985-1988) from the Ross Desert (McMurdo Dry Valleys) cryptoendolithic microbial ecosystem. *Antarctic Res. Series* 61, 201-207.

McKay, C.P., Molaro, J.L., and Marinova, M.M. (2009). High-frequency rock temperature data from hyper-arid desert environments in the Atacama and the Antarctic Dry Valleys and implications for rock weathering. *Geomicrobiology* 110, 182-187.

Moran, M.A., and Miller, W.L. (2007). Resourceful heterotrophs make the most of light in the coastal ocean. *Nature* 5, 792-800.

Naganuma, T., Otsuki, A. and Seki, H. (1989). Abundance and growth rate of bacterioplankton community in hydrothermal vent plumes of the North Fiji Basin. *Deep Sea Res. 3*6, 1379-1390.

Nealson, K.H. (1997). Sediment bacteria: Who's there, what are they doing, and what's new? *Annu. Rev. Earth Planetary Sci.* 25, 403-434.

Newberry, C.J., Webster, G., Cragg, B.A., Parkes, R.J., Weightman, A.J., and Fry, J.C. (2004). Diversity of prokaryotes and methanogenesis in deep subsurface sediments from the Nankai Trough, Ocean Drilling Program Leg 190. *Environ. Microbiol.* 6, 274-287.

Omelon, C.R., Ferris, F.G., Pollard, W.H., Andersen, D.T., and Whyte, L. (2003). "High Arctic cryptoendolithic microorganisms in a polar desert environment," in Permafrost. ed. Philips, Springman, Anderson. Lisse, Swets & Zeitlinger, 851-856

Oren, A. (2006). "Prokaryotic life." in Life as we know it. Ed. J. Seckbach. Dordrecht, Springer, 21-35

Protasov, A.A. (2008). River and Lake continua: an attempt at analysis and synthesis. *Inward Water Biol.* 1, 105-113.

Purdy, K.J., Nedwell, D.B., and Embley, T. M. (2003). Analysis of the sulfate-reducing bacterial and methanogenic archaeal populations in contrasting Antarctic Sediments. *Appl. Environ. Microbiol*. 69, 3181-3191.

de los Rios, A., Wierzchos, J., Sancho, L.G., and Ascaso, C. (2003). Acid microenvironments in microbial biofilms of Antarctic endolithic microecosystems. *Environ. Microbiol.* 5, 231-237.

Schwartz, J.I.K., Eckert, W., and Conrad, R. (2007). Community structure of Archaea and Bacteria in a profundal lake sediment Lake Kinneret (Israel). *Syst. Appl. Microbiol.* 30, 239-254.

Sherr, E.B., and Sherr, B.F. (2009). Capacity of herbivorous protists to control initiation and development of mass phytoplankton blooms. *Aquatic Microbial Ecol.* 57, 253-262.

Sun, H. J., and Friedmann, E. I. (1999). Growth on geological time scales in the antarctic cryptoendolithic microbial community. *Geomicrobiol. J.* 16, 193-202.

Takai, K., and Horikoshi, K. (1999). Genetic diversity of archaea in deep-sea hydrothermal vent environments. *Genetics* 152, 1285-1297.

Takai, K., Komatsu, T., Inagaki, F., and Horikoshi, K. (2001). Distribution of Archaea in a Black Smoker Chimney structure. *Appl. Environ. Microbiol.* 67, 3618-3629.

Takai, K., Oida, H., Suzuki, Y., Hirayama, H., Nakagawa, S., Nunoura, T., Inagaki, F., Nealson, K.H., and Horikoshi, K. (2004). Spatial distribution of marine Crenarchaeota Group I in the vicinity of deep-sea hydrothermal systems. *Appl. Environ. Microbiol.* 70, 2404-2413.

Takami, H., Inoue, A., Fuji, F., and Horikoshi, K. (1997). Microbial flora in the deepest sea mud of the Mariana Trench. *FEMS Microbiol. Lett.* 152, 279-285.

Tao, L, Peng, W., and Pinxian, W. (2008). Microbial diversity in surface sediments of the Xisha Trough, the South China Sea. *Acta Ecol. Sinica* 28, 1166-1173.

Tittel, J., Bissinger, V., Zippel, B., Gaedke, U., Bell, E., Lorke, A. and Kamjunke, N. (2003). Mixotrophs combine resource use to outcompete specialists: Implications for aquatic food webs. *Proc. Natl. Acad. Sci. USA* 100, 12776-12781.

Teske, A., and Sorensen, K. B. (2008). Uncultured archaea in deep marine subsurface sediments: have we caught them all? *ISME J.* 2, 3-18.

Walker, J.J., Spear, J.R., and Pace, N.R. (2005). Geobiology of a microbial endolithic community in the Yellowstone geothermal environment. *Nature* 434, 1011-1014.

Walker, J.J., and Pace, N.R. (2007). Endolithic microbial ecosystems. *Annu. Rev. Microbiol.* 61, 331-347.

Waragai, T. (1998). Effects of rock surface temperature on exfoliation, rock varnish, and lichens on a boulder in the Hunza Valley, Karakoram Mountains, Pakistan. *Arctic Alpine Res.* 30, 184-192.

Wierzchos, J., Ascaso, C., and McKay, C. P. (2006). Endolithic cyanobacteria in halite rocks from the hyperarid core of the Atacama desert. *Astrobiology* 6, 415-422.

Xiangchum, Q., Yulai, W., Weicong, X., Mengchang, H. and Chunye, L. (2009). Distribution of microbial community in the sediments of the Daliao River watersystem, China. *Environ. Earth Sci.* 61, 1725-1734.

Yang, Y., Itoh, T., Yokobori, S., Itahashi, S., Shimada, H., Satoh, K., Ohba, H., Narumi, I., and Yamagishi, A. (2009). *Deinococcus aerius sp. nov.*, isolated from the high atmosphere. *Intl. J. Syst. Evol. Microbiol.* 59, 1862-1866.
