# Supplementary material for: A General Framework of Persistence Strategies for Biological Systems Helps Explain Domains of Life
Source: Front Genet. 2013 Feb 25;4:16. doi: 10.3389/fgene.2013.00016 (PMC3580334; doi:10.3389/fgene.2013.00016)
Supplement: Supplementary Datasheet S4 — Methods. [file 36588_Caetano-Anolles_DataSheet4.PDF]

Additional file 4

METHODS

General data. The data used to make statements about scope, i.e. body size, speed, lifespan, and spatial range, were obtained from primary literature, and were listed in the Additional File 1 as published, with the respective references. We strove to find data for concrete species. When it was not possible, trends were completed with data for groups of organisms, such as "caterpillars" or "cephalopods". The data are inhomogeneous: some numbers corresponded to the mean measured value (e.g. mean length), some were listed in the publications as being "characteristic of the species", and for some only the range was known (and we took the average). We do not believe this hampers our study, since we do not perform numerical analysis on these data. The scope variables were used to illustrate the applicability of our concepts to extant organisms from the six kingdoms. The trends displayed in our figures are valid and sufficient for our purposes.

Organism size. The data on linear organism size were obtained from literature and are listed in Additional File 3. For organisms analyzed the longest linear dimension of the body was always chosen. For some groups of organisms, e.g. bumblebee or nematodes, precise measurements were not found, but good estimations were available from the experts. In those cases the numbers were listed as approximate (e.g. ~1 mm).

Mycelial fungi principally do not have a "characteristic body size". Therefore we gathered fungal data to capture the range of growth patterns for Figure 5. Apical hyphal length represented the size of the actively growing component of the mycelium. Mycelium diameter, where available, represented the total body size in the exponential growth phase. Species of *Armillaria* were used specially to probe growth patterns of this largest known fungus: from the invasive rhizomorphs to the entire genet.

All plant sizes were given for mature adult state, even though some of the growth speed data were provided for seedling roots. We strove to display the trends for spatio-temporal scale characteristic of organisms, including the borderline cases of the biggest, longest-living and the fastest organisms. Seedling root tips were used to illustrate a fast-growing stage of plant lifecycle, but the organism size was given for the adult, because that is what defines the spatial scale characteristic of the individual, and to which the juvenile growth ultimately leads. Linear sizes of non-clonal species were given as height of an individual including the inflorescence. Linear sizes of clonal species were given as diameter of a genet, since their lateral growth frequently exceeds their height. These above-ground measurements were given even for the data illustrating root growth, because the size of root systems is difficult to measure, and few data are available.

Due to the great variety of shapes and sizes of organisms in Akarya and Protista, their respective vertical bars on Figure 2 mark the approximate values of body size, not the measured average. In contrast, we used the measured dimensions of fully sequenced unicells to calculate cell volumes displayed on Figure 4. Those data were obtained from published measurements, by approximating from published micrographs or from published "ballpark" estimations by authors known to be dedicated students of the organism. It was impossible to find precise measurements

of cell dimensions for all fully sequences unicellular organisms. However, anecdotal data were not used. If a trusted source of measurement or estimation could not be found, the entry was left blank in the Additional file 3.

Data for cell volumes of fully sequenced multicellular organisms displayed on Figure 4 were less precise than unicellular organisms. Approximate calculations were sometimes made, such as for nematodes, based on the organism's total volume, and the known total number of cells in the organism. For vertebrates, we took liver cells as representatives of the "generic cell", and attempted to find references to smallest and largest cells characteristic of the species. For some organisms, data from a very closely related species were used in absence of direct measurements. This was clearly noted in Additional file 3. For fungi, we used measurements of basal, not apical cells whenever possible. Apical cells are constantly growing and dividing, and their size cannot be used as characteristic of the species. In multinucleate fungi, volume was calculated as that of the entire cell, divided by the characteristic number of nuclei. For dimorphic fungal species, hyphal dimensions were used, since hyphal mycelia represent the state induced by favorable environmental conditions.

Spatial range. The spatial ranges of unicellular organisms are difficult to measure, and are given in our datasets only approximately, as a guideline for comparison to other kingdoms. Body size, genet diameter and mycelium diameter was used for spatial range of sessile plants and fungi. Home range measurements were used to characterize the spatial range of most metazoa, as they are readily available for many species. It is an underestimation, since "occasional sallies, perhaps exploratory in nature, should not be considered as part of the home range" (Burt 1943). However, our definition of spatial scope encompasses the greatest expanse of movement of the animal individual under consideration.

Motility speed. Because an individual's spatial scope size depends greatly on the motile capabilities of the individual, we made the effort to illustrate the extent of evolution of motility for each kingdom. Thus, we listed as many known types of locomotion as possible, and attempted to present data covering the entire range of speeds, from slowest to fastest known species in each kingdom. These properties were clearly marked in the Additional files 1.

Because plants and fungi are sessile, their root, shoot, branch and hyphae elongation rates were used to estimate the "motility speed", even though the term does not strictly apply to these two kingdoms. We attempted to present data from a variety of species with substantially differing growth speeds. Among the fungi we cover the range from the fast-growing, opportunistic *Neurospora*, to the long-lived, slow-growing *Armillaria*. Among the plants we cover the range from Bamboo – possibly the fastest-growing plant on Earth – to desert xerophytes.

Life span. The lifespan of unicells was recorded as time between divisions. For fungal species that senesce, the lifespan was estimated as time-to-senescence. For immortal fungi we sampled the data on hyphal septation times and mycelium dry weight doubling time. These data are not indicative of mycelium longevity, but they help estimate the temporal scale of its constituents: hyphae. This is useful, considering that a single hypha can independently establish a new mycelium upon fractionation. The estimated age of the famous Oregon *Armillaria* mycelium, the longest-living known fungal mycelium, was used as an upper bound on the fungal longevity.

Longevity for annual and biennial plants was estimated based on the characteristic length of the growth season in temperate climates. The data for longevity of monocarpic perennials were found in the literature. Polycarpic perennials, in principle, do not senesce (Sklensky and Davies 1993, Gan 2007), but lifespan of individuals has been recorded in the literature for some species, based on the age at the time of death due to disease or physical damage due to weather. These numbers underestimate longevity of perennial plants. Longevity of metazoan species was found in the literature and recorded as published.

Genetic and protein architecture data. Statistics on protein superfamilies content of the fully sequenced genomes were obtained from the SUPERFAMILY 1.69 database (Gough et al. 2001). The dataset includes a total of 877 folds and 1453 FSF cumulatively present in 584 organisms with fully sequenced genomes, including 46 Archaea, 397 Bacteria and 141 Eukarya (Additional file 3). This was the complete set of fully sequenced organisms in February 2008. While this number has been tripled by 2011, the dataset was sufficiently large to allow us to demonstrate the trends that illustrate our theory. Those trends are unlikely to change in an expanded dataset.

Determination of lifestyles. Lifestyles of fully sequences organisms were manually annotated as free-living (FL), parasitic (P), obligate parasitic (OP), using various sources of information, mostly primary research papers, books and online databases (Additional file 3). Thus, the entire dataset contains 105 eukaryotes, 225 bacteria and 44 archaea that are free-living organisms, 17 eukarya, 60 bacteria and 1 archaeum that are facultative parasites, and 19 eukarya, 112 bacteria and 1 archaeum that are obligate parasites.

In many cases the exact statement of an organism's lifestyle could not be found, but could be inferred from the description of behavior, ecology and nutritional requirements. The following guidelines were used to help with decisions. An organism was defined as OP if its lifecycle could not proceed without a host. This could mean inability to survive, or grow, or reproduce, or any combination of the above. An organism was defined as P if it could undergo its natural lifecycle independently of other specific organisms, but opportunistically made use of cellular organization of other species. An organism was defined as a P or OP, as opposed to FL, if it recruits the specific host's intra- or intercellular resources for its own needs. An organism which is a pathogen due to secretion of substances toxic to the host, but which does not use the host's cellular organization, is defined as free-living. For example, *Bacillus cereus* is a soil-dwelling saprophytic organism that secretes enterotoxins, causing diarrhea, nausea and vomiting. These enterotoxins are a part of normal secretions of the organism, which do not specifically target the animal that happened to ingest the bacterium's endospores. The bacillus feeds on the products of decay of organic matter; intestinal tract of animals will contain such products, so that the ingested endospores can germinate, and bacilli will subsequently proliferate, as they would normally in soil. We consider this germination and feeding on the host's feces as opportunistic use of suitable environment, not a parasitic adaptation that evolved for exploitation of the host, although the latter is possible. In contrast, if an organism, e.g. *Clostridium tetani*, displays ability to secrete specific toxins with a very host-dependent mechanism of uptake and action, directed at killing the target cell and reproducing in it or in products of its destruction, it is defined as P.

In the absence of data on the mechanism of pathogenicity of a pathogenic organism, it was defined as P, since no support for OP nature was found. A commensal organism that could not be

confirmed as OP or FL, was defined as P regardless of its potential for pathogenicity. Organisms that are facultative intracellular pathogens with very specialized adaptations for vector transfer and host adhesion (e.g. *Bartonella bacilliformis*) are defined as OP.

Errors were inevitable during this data acquisition. The authors recognize that for many parasitic organisms, OP vs. P assignments were done incorrectly. The reader should take these assignments with a grain of salt. The general relationships and conclusions made in this paper will stand in spite of some uncertainty in P/OP distinction.

Given the time-consuming nature of such search for data, we allowed ourselves to interpolate data for organisms that belonged to closely related physiological groups, such as *Lactobacilli*, *Staphylococci*, etc., unless exceptions were found in the literature. Every effort was made to make the dataset as complete as possible. However, it is the general trends that are important for this study, and those trends would not likely be affected by such interpolations, since the respective datapoints tend to form fairly tight clusters. All sources of data and the interpolations are clearly noted in the Additional File 3.

Environmental parameters. The viability ranges for temperature, salinity and pH characteristic of the 584 fully sequenced organisms were found in primary literature. When the range is only known for optimum growth, those numbers were used to approximate the viability range. For some species data were approximated from the genus.

When only the conditions of no growth were found, the viable ranges of temperature, pH and salinity were determined approximately, with precision appropriate for the variable. For example, if published data indicated that an organism does not grow at temperatures below a°C and above b°C, then viable temperatures were assumed to be from a+1°C to b-1°C. Likewise, pH between a+0.1 and b-0.1, and salinity between a+0.1%w/v and b-0.1%w/v were used. If minimum salinity could not be found, it was assumed to be 0% w/v – the absolute minimum possible. If published data indicated an organism's characteristic habitat to be seawater, freshwater or brackish water, characteristic salinity was assumed to be at 3.5, 0.8 and 0% w/v, respectively. If an organism was known to have "no obligate salt requirement", characteristic salinity was assumed to be of 0% w/v.

All intracellular parasites of mammals were assumed to have an optimum temperature of 37°C, pH of 7 and salinity characteristic of the invaded cell type. Extracellular parasites of mammals had similar characteristics, except salinity was listed as 0.8% (assuming extracellular NaCl of 140mM).

Animals and plants were assumed to grow best at 20°C, and so were obligate parasites of plants, except potato blight known to grow best at 10°C (see bacterial data file for references). From published lab culture experiments, fungi were approximated to grow best at 25°C.

Analysis of the molecular functions of domain combinations associated with the P-loop hydrolase domain. UniProt sequences harboring the P-loop hydrolase FSF (c.37.1) and a total of 134 domain combinations that included this domain were annotated with GO terms of molecular function (*mf*, level 2) by making use of Gene Ontology Annotation (GOA) (GOA

2011) resources. Protein sequences were assigned to structures using Hidden Markov Models (HMMs) of structural recognition (Wang and Caetano-Anollés 2009). Sequences with long non-domain regions (> 30 residues) were excluded, making it unlikely that a domain or transmembrane region be present in inter-domain regions. Structures were mapped to a published timeline of protein domain discovery at FSF level of structural abstraction (Wang and Caetano-Anollés 2009) and the accumulation of structures and GO level 2 functions plotted against FSF age (nd_{FSF}).

Principal Coordinate Analysis and use/reuse plots of FSFs in proteomes. Similarities (dissimilarities) that exist between FSF repertoires in proteomes were explored using multivariate statistics with Principal Coordinate Analysis (PCoA) (Gower 1966). PCoA is a complementary procedure to clustering and commonly utilized in ecological and genomics studies (Legendre and Legendre 1998). It enables graphical visualization of objects (proteomes) on each of several axes (one for each variable i.e., FSF) such that objects (proteomes) similar to each other are nearer and dissimilar are farther apart. We generated the presence/absence matrix for 1,733 FSFs defined by the Structural Classification of Proteins (SCOP) version 1.75 (Murzin et al. 1995) that were present in a dataset of 903 proteomes corresponding to 70 Archaea, 601 Bacteria, 93 Fungi, 29 Protista, 24 Plants and 86 Metazoa. We then calculated a similarity matrix for proteomes based on Pearson correlations using XLSTAT, an add-in to Microsoft Excel (XLSTAT 2011). This similarity matrix was given as input to the PCoA function, which returns a series of eigenvectors. These eigenvectors correspond to the principal coordinates that can be used to display proteomes in a multi-dimensional space. We visualized group differences in 3-dimensional space using OriginPro (OriginPro 2011) The distances between objects (proteomes) on the 3-dimensional plot approximately represent actual similarities between proteomes (Jolliffe 2002).

We plotted the FSF use (total number of distinct FSFs in a proteome defined by FSF diversity) versus reuse (total number of FSFs in a proteome defined by FSF abundance) to describe a congruent trend of organisms towards structural complexity in a set of 591 free-living organisms. Values were log transformed to make scales easy to interpret.

Phyla coverage in the dataset of fully sequenced organisms. It is known that sequencing projects are biased in their choice of organisms, by considerations of usefulness for medical or research purposes. Therefore, the organismal phyla are not represented uniformly in our dataset. The Additional File 5 contains phyla coverage data within the fully sequenced Akarya used in this study. Organisms were determined to belong to the phyla according to PUBMED taxonomy database (PUBMED 2009), accessed in October 2009.

REFERENCES

Burt, W. H. (1943). Territoriality and home range concepts as applied to mammals. *J. Mammalogy* 24, 346-352.

Gan, S. (2007). Senescence processes in plants. Oxford: Blackwell Publishing Ltd.

Gough, J., Karplus, K., Hughey, R., and Chothia, C. (2001). Assignment of homology to genome sequences using a library of hidden markov models that represent all proteins of known structure. *J. Mol. Biol.* 313, 903-919.

Gower, J.C. (1966). Some distant properties of latent root and vector methods used in multivariate analysis. *Biometrika* 53, 325-38.

Jolliffe, I.T. (2002). Principal component analysis. New York: Springer-Verlag.

Legendre, P., and Legendre, L. (1998). Numerical ecology. Elsevier science: Amsterdam.

Murzin, A.G., Brenner, S.E., Hubbard, T., and Chothia, C. (1995). SCOP: a structural classification of proteins database for the investigation of sequences and structures. *J. Mol. Biol.* 247, 536-540.

OriginPro (2011). Originlab data analysis and graphing software. http://www.originlab.com/index.aspx?go=Products/OriginPro

PUBMED taxonomy (2009). http://www.ncbi.nlm.nih.gov/taxonomy

Sklensky, D.E., and Davies, P.J. (1993). "Whole plant senescence: reproduction and nutrient partitioning," in Horticultural Reviews, Volume 15, ed. J. Janick. Wiley & Sons, Inc, 336-366

Wang, M., and Caetano-Anollés. G. (2009). The evolutionary mechanics of domain organization in proteomes and the rise of modularity in the protein world. *Structure* 17, 66-78.

XLSTAT (2011). Adinsoft. http://www.xlstat.com/en/
